# Combinatorial analysis of *ACE* and *ACE2* polymorphisms reveals protection against COVID-19 worsening: A genetic association study in Brazilian patients

**DOI:** 10.1371/journal.pone.0288178

**Published:** 2023-11-30

**Authors:** Romes Bittencourt Nogueira de Sousa, Lis Raquel Silva do Nascimento, Luiz Henrique Alves Costa, Vanessa Rafaela Milhomem Cruz Leite, Clayton Luiz Borges, José Miguel de Deus, Ana Cristina Silva Rebelo, Denise da Silva Pinheiro, Gustavo Rodrigues Pedrino

**Affiliations:** 1 Department of Physiological Sciences, Institute of Biological Sciences, Federal University of Goiás (UFG), Goiânia, Goiás, Brazil; 2 Laboratory of Molecular Biology, Institute of Biological Sciences, Federal University of Goiás (UFG), Goiânia, Goiás, Brazil; 3 Department of Gynecology and Obstetrics, Federal University of Goiás, Goiânia, Goiás, Brazil; 4 Department of Morphology, Institute of Biological Sciences, Federal University of Goiás (UFG), Goiânia, Goiás, Brazil; 5 Laboratory of Clinical Analysis and Health Education, Institute of Biological Sciences, Federal University of Goiás (UFG), Goiânia, Goiás, Brazil; Osaka City General Hospital, Children’s Medical Center, JAPAN

## Abstract

Since angiotensin-converting enzyme 2, ACE2, was identified as the receptor for SARS-CoV-2 and considering the intense physiological interplay between the two angitensinases isoforms, ACE and ACE2, as counter-regulatory axis of the renin-angiotensin system, we proposed the evaluation of polymorphisms in these two key regulators in relation to COVID-19 severity. A genetic association study involving 621 COVID-19 hospitalized patients from Brazil was performed. All subjects had a confirmed diagnosis of COVID-19 via RT-PCR. Patients were categorized into two groups: the "mild" group (N = 296), composed of individuals hospitalized in ward beds who progressed to cure, and the "severe" group (N = 325), composed of individuals who required hospitalization in an intensive care unit (ICU), or who died. Blood samples were genotyped for ACE I/D polymorphism and ACE2 G8790A polymorphism by real-time PCR via TaqMan assay. The analysis of combined polymorphisms revealed a protective role for genotypic profile II/A_ (OR^A^ = 0,26; p = 0,037) against the worsening of COVID-19 in women. The results indicate a protection profile to COVID-19 progression, in which the II/A_ carriers have almost four times less chance of a severe outcome. It is proposed that a decreased activity of ACE (deleterious effects) in conjunction with an increased ACE2 activity (protective effects), should be the underlying mechanism. The findings are unprecedented once other studies have not explored the genotypic combination analysis for ACE and ACE2 polymorphisms and bring perspectives and expectations for dealing with the COVID-19 pandemic based on definitions of genetically-based risk groups within the context of personalized medicine.

## Introduction

Coronavirus 19 Disease (COVID-19) is a condition that has been associated with pathophysiological impairment of the Renin-Angiotensin System (RAS) as a result of infection with the viral agent SARS-CoV-2 [1–4]. Virus entry into the intracellular environment involves the sequestration of Angiotensin-Converting Enzyme 2 (ACE2), an angitensinase identified as the receptor of the new coronavirus, in the membrane of target cells [2, 5–7]. This process, which has made ACE2 one of the most studied proteins in recent times [8], can directly lead to RAS imbalance affecting several RAS components, which may be the pivotal link of the new coronavirus with acute lung injury and multiorgan failure [9–12]. The key role played by angitensinases, ACE2 and its homolog ACE, in the regulation of the RAS, has led researchers to launch a hypothesis regarding genetic susceptibility to COVID-19 involving polymorphisms that alter the activity and/or expression of these enzymes, which has been confirmed in some studies.

With the invasion of the coronavirus, ACE2 undergoes downregulation, a process involving viral attachment, internalization together with viral particles, and subsequent degradation, resulting in ACE2 depletion from the membrane of the host cells [6–8, 13]. In addition to the internalization of ACE2 upon viral attachment, activation of ADAM17 (a disintegrin and metalloproteinase domain-containing protein 17), also contributes to the decline in ACE2 expression on cell membranes, with the generation of circulating soluble ACE2 [14]. In this sense, the rise in Ang II in COVID infection reflects its reduced biotransformation to Ang 1–7 due to declining ACE2 expression on endothelial cell membranes, which triggers acute deterioration of the lung and other tissues due to alterations such as inflammation, oxidative stress, fibrosis, and thrombosis [5, 10, 11, 14, 15]. In this way, this may be one of the main mechanisms involved in the pathophysiology of coronaviruses, particularly SARS-CoV-2, which still demands better scientific elucidation [14, 16].

Therefore, physiological profiles of ACE and ACE2 must be associated with the outcome of patients affected by COVID-19 by the impairment of angiotensin II to angiotensin 1–7 balance [7, 12]. Corroborating this line of evidence, patients with previous decreased ACE2 activity, such as in the elderly, systemic arterial hypertension (SAH), diabetes mellitus (DM), and cardiovascular diseases (CVDs), are more subject to longer hospitalizations and death from COVID-19, that is, tend to worsen the disease, and such conditions are considered risk factors [1, 3, 17–19].

The literature has demonstrated the association between polymorphic variations in the *ACE* (chromosome 17q23.3) and *ACE2* (chromosome Xp22) genes and susceptibility to cardiometabolic diseases such as SAH, DM, and CVDs [20–22]. In this context, *ACE* I/D (rs2106809) and *ACE2* G8790A (rs2285666) polymorphisms can be highlighted as the most studied due to their effect on the functionality of the enzymes by impacting genetic mechanisms of alternative splicing (*ACE2* G8790A polymorphism in intron 3) or even for presenting linkage disequilibrium with several other genetic polymorphisms (*ACE* I/D polymorphism in intron 16) [23–29]. In this sense, it is reasonable to infer that those polymorphic changes could also affect the prognosis of patients with COVID-19, with the D allele of *ACE*, associated with increased enzyme activity, and the *ACE2* G allele, associated with decreased enzyme activity, prone to deleterious effects in the organisms, as previously shown in a study of our research group related to hypertension susceptibility [22].

In this regard, considering the recent literature, Srivastava *et al*. (2020) [30] demonstrated the correlation of the A allele of *ACE2* G8790A polymorphism with a lower rate of infection and lethality (r = −0.699; p = 0.002) in an ecological study involving Indian populations affected by the disease. Möhlendick *et al*., (2021) [31] have obtained an association between the G allele of *ACE2* G8790A polymorphism and the risk for SARS-CoV-2 infection and the clinical course of COVID-19 in a case-control study conducted in the German population, while the *ACE* I/D polymorphism had no association. Karakaş Çelik et al. (2021) [32] did not find an association between both polymorphisms and severity in patients affected by COVID-19 in a case study performed in the Turkish population.

The divergent results among the studies cited imply the need for further research involving different populations, and it is noteworthy that the genotypic combination analysis of the two RAS components was not explored, as proposed in the present study, to achieve the provable interplay between them in disease outcome. The current study sought to assess the impact of ACE and ACE2 polymorphisms on the prognosis of COVID-19 patients with a confirmed diagnosis of SARS-CoV-2 infection being treated in a field hospital in Goiânia, GO, Brazil. The results indicated, for the first time in the literature, a genetic protection factor for the susceptibility to the worsening of COVID-19, which was mediated by the combined polymorphisms of ACE and ACE2. We should also highlight that this is the first study to investigate a genetic association in the outcome of COVID-19 carried out in the Brazilian population.

## Material and methods

### Ethical statement

This study was approved by the Ethics Committee of the Federal University of Goiás (4140331/2020). It was conducted in accordance with the ethical principles for medical research involving human subjects of the World Medical Association Declaration of Helsinki, and, after explaining the research, the volunteers or their families signed a free and informed consent form.

### Subjects

A total of 621 individuals (348 men and 273 women, age = 61.45±15.90 years) were recruited during the period from August to December 2020, while SARS-CoV-2 were the prevalent circulating serotype of Coronavirus in Brazil and before the beginning of vaccination [33]. Patients were subdivided into the "moderate group" (n = 296, 161 men, 135 women, age = 57.49±15.78 years) and the "severe group" (n = 325, 187 men, 138 women, age = 65, 06±15.20 years). All subjects were diagnosed with COVID-19 by RT-PCR. The moderate group was composed of patients who received health care at the Municipal Field Hospital of Goiânia-Goiás, and who were admitted to a hospital bed in a ward, having evolved to cure. The severe group consisted of those who required admission to the Intensive Care Unit (ICU) in the same hospital, as previously informed, including individuals who died while hospitalized in ICU beds.

Sociodemographic and clinical data (gender, age, and comorbidities) were collected by consulting medical records, in which the daily evolution of each patient was sought to allow discrimination between the research groups. Comorbidities were grouped into the following subgroups, according to the etiology of the disease or disorder (only data of preceding diseases were collected): hypertension, diabetes, respiratory system diseases (RSDs), cardiovascular diseases (CVDs), obesity, neuropsychiatric diseases, and disorders (NDDs) and acute or chronic kidney diseases (ACKD). Data on smoking was also collected. All these data were confirmed and considered in the statistical analyses for comparison between groups in the regression modeling.

Peripheral blood samples were collected through venipuncture in 4 ml ETDA tubes and centrifuged at 5000 rpm for 20 minutes to allow separation of the leukocyte ring. This was collected by Pasteur pipettes and stored at -20°C in 1.5 ml microtubes, aiming at DNA extraction.

### Genotyping of ACE and ACE2 polymorphisms

DNA extraction was performed using the DNA PureLink™Genomic DNA Mini Kit, according to the manufacturer’s suggestions. The extraction products were labeled and stored at -80o C for use in the patronized genotyping reaction. Molecular analysis of genetic polymorphisms was performed by a real-time polymerase chain reaction (qPCR) TaqMan assay.

In the genotyping of *ACE* I/D polymorphism, it was adopted the protocol developed by Koch et al. [34] adapted for use in the QuantStudio 5 (Thermofiher®) apparatus. Primers sequences were: ACE111: 5’-CCCATCCTTTCTCCCATTTCTC-3’; ACE112: 5’-AGCTGGAATAAAATTGGCGAAAC-3’; ACE113: 5’-CCTCCCAAAGTGCTGGGATTA-3’. Probes sequences were: VIC-5’-AGGCGTGATACAGTCA-3’ (I allele); FAM-5’-TGCTGCCTATACAGTCA-3’ (D allele).

Thermocycling conditions were: Pre-Hold—60°C for 30 seconds; Hold-Stage—95°C for 10 minutes; PCR-Stage (40 cycles) - 95°C for 15 seconds and 60°C for 1 minute; Post-Read Stage—60°C for 30 seconds.

The discrimination of genotypes II, ID, and DD was performed by analyzing the amplification curves at the end of thermocycling. It was considered: Allele 1- VIC–Insertion (ACE111/ACE113-ACE112); Allele 2- FAM–Deletion (AC111-AC112).

For the genotyping of ACE2 G8790A SNP polymorphism, a previously developed and validated TaqMan SNP allelic discrimination assay was adopted (ThermofisherScientific®, Assay ID: C__2551626_1; SNP ID: rs2285666) for use in the QuantStudio 5 device. The thermocycling conditions were the same as previously described for ACE I/D. Allelic discrimination, as well as the sequence of probes and primers, followed the protocol established by Thermofisher®. The notation A_ is used to refer to AA and GA genotypes, while G_ is used to designate GG and GA genotypes.

### Statistical analysis

Sociodemographic, clinical, and genotyping data were tabulated in Microsoft Office Excel 2007 software and analyzed with BioEstat v. 5.3 (Mamiraua Institute, available at https://mamiraua.org.br) and IBM SPSS v. 22. For comparison between groups, continuous variables were expressed as mean and standard deviation and analyzed using the T-test, while qualitative variables, expressed as yes/no, were analyzed using the χ2 test or Fisher’s exact test when necessary. To analyze the susceptibility to COVID-19 outcomes (moderate and severe groups), the Odds Ratio (OR) test was performed, with a 95% confidence interval, and adjusted Odds Ratio (OR^A^) values were obtained by binary multifactorial logistic regression analysis, with control of confounding factors. The confounding factors used were age, hypertension, diabetes, respiratory system diseases, cardiovascular diseases, obesity, neuropsychiatric diseases and disorders, acute or chronic kidney diseases and smoking (Table 1). In relation to the genotypes, the fidelity to the Hardy-Weinberg Equilibrium was measured by the χ2 test. The *p-value* considered indicative of significance was p<0.05. As ACE2 gene is located on the X chromosome, the multinomial logistic regression was performed separately for the two sexes where it appears.

**Table 1:** Variables in the multifactorial logistic regression analysis for ACE I/D and ACE2 G8790A polymorphisms in the moderate and severe COVID-19 groups.

Variables on Regression Modeling	Analyzes in the Study
Age	All Analyses of Logistic Regression
Sex	Table 3 –ACE I/D Polymorphism
Comorbidities (Hypertension, Diabetes, RSDs, CVDs, Obesity, NDDs, ACKDs)	All analyses of Logistic Regression
Smoking	All analyses of Logistic Regression
ACE And ACE2 Genotyping Data	According To Each Analysis as Below Listed
• ACE I/D Genotypes	Table 3 –ACE Polymorphism
• ACE I/D Additive Model Genotypes	Table 3 –ACE Polymorphism
• ACE2 G8790A Genotypes for Females	Table 3 –ACE2 Polymorphism in Females
• ACE2 G8790A Additive Model Genotypes for Females	Table 3 –ACE2 Polymorphism in Females
• ACE2 G8790A Alleles for Males	Table 3 –ACE2 Polymorphism in Males
ACE/ACE2 Dominant Model Genotypes for Females	Table 4 - ACE/ACE2 Dominant Model in Females
ACE/ACE2 Recessive Model Genotypes for Females	Table 4 - ACE/ACE2 Recessive Model in Females
ACE/ACE2 Genotypes for Males	Table 4 - ACE/ACE2 in Male

RSDs–Respiratory System Diseases. CVDs–Cardiovascular Diseases. NDDs–Neuropsychiatric Diseases or Disorders. ACKDs–Acute or Chronic Kidney Diseases.

## Results

Clinical and sociodemographic variables for the studied groups are shown in Table 2. The distribution by sex, obesity and smoking variables did not show disagreement between the groups, pointing to homogeneity for the subsequent analysis. As expected, the variable age had increased values in the severe outcome group (p<0.001), once it was a considerable risk factor for the worsening of patients affected by COVID-19. The same occurred for all the clinical conditions and diseases evaluated, which were significantly more frequent in the severe group, corroborating the well-known role of comorbidities in disease aggravation.

**Table 2:** Characterization of clinical variables for the moderate and severe groups.

Variables	Moderate Group (N = 296)	Severe Group (N = 325)	*p*
Age (years)	57,49±15,78	65,06±15,20	<0,001*
Sex (M/F)	161/135	187/138	0,4301
Hypertension (Y/N)	106/190	144/181	0,0310*
Diabetes (Y/N)	49/247	88/237	0,0016*
RSDs (Y/N)	33/263	66/259	0,0018*
CVDs (S/N)	18/278	49/276	0,0003*
Obesity (Y/N)	15/281	29/296	0,0614
NDDs (Y/N)	16/280	48/277	0,0001*
ACKDs (Y/N)	2/294	20/305	0,0002*
Smoking (Y/N)	25/271	32/293	0,5461

Analysis by Student’s T test or χ^2^ Test. *Significant difference between groups (p < 0.05). RSDs–Respiratory System Diseases. CVDs–Cardiovascular Diseases. NDDs–Neuropsychiatric Diseases or Disorders. ACKDs–Acute or Chronic Kidney Diseases. Y–Yes. N—No

Regarding the genotypic analysis (Table 3), in the moderate group, the frequencies were: ACE I/D: II: 19.6%; ID: 48.0%, DD: 32.4%. For the ACE2 G8790A polymorphism, in females, the frequencies were: 50.4% for GG, 38.5% for GA, and 11.1% for AA. The allelic frequencies for males were 76.4% (G) and 23.6% (A). In the severe group, the following values were observed for ACE I/D polymorphism: 20.3% for II; 53.5% for ID, and 26.8% for DD. For the ACE2 G8790A polymorphism, in females, the frequencies were: 58.7% for GG, 34.8% for GA, and 6.5% for AA. Allelic frequencies for males were: 78.1% (G) and 21.9% (A). No statistically significant differences were observed in the frequency distribution between groups (analyses by the chi-square test), and no association was verified in the susceptibility analysis for each genetic polymorphism individually.

**Table 3:** Genotypic frequency distribution of ACE I/D and ACE2 G8790A polymorphisms in the moderate and severe COVID-19 groups and risk analysis for the outcome in COVID-19.

Genotype	Moderate Group (%)	Severe Group (%)	χ^2^	*p* ^1^	OR (95%CI)	OR^A^ (IC95%)	*p* ^2^
**ACE**							
Total	296 (100)	325 (100)					
II	58 (19,6)	66 (20,3)	-------	-------	1 (Reference)	1 (Reference)	---------
ID	142 (48,0)	172 (52,9)	0,04	0,852	1,06 (0,70–1,61)	1,14 (0,73–1,78)	0,577
DD	96 (32,4)	87 (26,8)	0,74	0,389	0,80 (0,50–1,26)	0,90 (0,55–1,47)	0,668
ID+DD	238 (80,4)	259 (79,7)	0,02	0,903	0,96 (0,64–1,42)	1,04 (0,68–1,59)	0,848
**ACE2**							
Female	135 (100)	138 (100)					
GG	68 (50,4)	81 (58,7)	-------	-------	1 (Reference)	1 (Reference)	---------
GA	52 (38,5)	48 (34,8)	0,73	0,392	0,77 (0,47–1,29)	0,75 (0,43–1,31)	0,313
AA	15 (11,1)	9 (6,5)	1,73	0,189	0,50 (0,21–1,22)	0,65 (0,25–1,67)	0,365
GA+AA	67 (49,6)	57 (41,3)	1,59	0,208	0,71 (0,44–1,15)	0,73 (0,43–1,23)	0,729
Male	161 (100)	187 (100)					
Allele G	123 (76,4)	146 (78,1)	-------	-------	1 (Reference)	1 (Reference)	---------
Allele A	38 (23,6)	41 (21,9)	0,06	0,807	0,91 (0,55–1,50)	0,84 (0,49–1,46)	0,541

Analysis by χ^2^ Test, calculation of *Odds Ratio* (OR) with confidence interval (95%CI), and Multinomial Logistic Regression to obtain adjusted Odds Ratio (OR^A^) values. *p^1^*—p value for the χ^2^ test or Fisher’s Exact Test. *p^2^*—p value for OR^A^. Significance level: p<0.05.

In the association analysis for the combined genotypes of the two genes (Table 4), there was a predominance of occurrence of the ACE/ACE2 genotypic profile II/A_ (p = 0.028) in the moderate group for females (analysis by the dominant model of ACE2 –grouping GA and AA individuals). The result was confirmed in the susceptibility analysis after adjustment by logistic regression, which revealed that it is a protective genotype against the worsening of COVID-19 (OR^A^ = 0.26; p = 0.037) conferring almost four times less chance of having a worse prognosis in the evolution of the disease. For the other genotypic combination models, no statistically significant differences were observed.

**Table 4:** Genotypic frequency distribution for the combination of ACE I/D and ACE2 G8790A polymorphisms in moderate and severe COVID-19 groups and risk analysis for the outcome in COVID-19.

Genotype	Moderate Group (%)	Severe Group (%)	χ^2^	*p* ^1^	OR (95%CI)	OR^A^ (IC95%)	*p* ^2^
***ACE/ACE2***							
**Female**	135 (100)	138 (100)					
***ACE/ACE2 Dominant Model***							
II/GG	16 (11,8)	23 (16,7)	----	------	1 (Reference)	1 (Reference)	-------
II/A_	15 (11,1)	5 (3,6)	4,83	0,028*	0,23 (0,07–0,77)	0,26 (0,08–0,92)	0,037*
ID/GG	23 (17,0)	34 (24,6)	0,02	0,884	1,03 (0,45–2,36)	0,95 (0,39–2,31)	0,916
ID/A_	33 (24,4)	38 (27,5)	0,12	0,726	0,80 (0,36–1,77)	0,76 (0,32–1,76)	0,515
DD/GG	29 (21,5)	24 (17,4)	1,18	0,277	0,58 (0,25–1,33)	0,52 (0,21–1,29)	0,156
DD/A_	19 (14,1)	14 (10,1)	1,35	0,245	0,51 (0,20–1,31)	0,48 (0,18–1,33)	0,158
***ACE/ACE2 Recessive Model***							
II/G_	26 (19,2)	27 (19,6)	----	------	1 (Reference)	1 (Reference)	------
II/AA	5 (3,7)	1 (0,7)	#	0,198	0,19 (0,02–1,76)	0,22 (0,02–2,17)	0,192
ID/G_	49 (36,3)	67 (48,5)	0,44	0,510	1,32 (0,69–2,53)	1,15 (0,56–2,33)	0,705
ID/AA	7 (5,2)	5 (3,6)	0,07	0,794	0,69 (0,19–2,44)	0,74 (0,20–2,82)	0,660
DD/G_	45 (33,3)	35 (25,4)	0,41	0,524	0,75 (0,37–1,50)	0,68 (0,31–1,46)	0,319
DD/AA	3 (2,2)	3 (2,1)	#	1,000	0,96 (0,18–5,21)	1,58 (0,25–9,97)	0,625
**Male**	161 (100)	187 (100)					------
***ACE/ACE2***							
II/G	21 (13,0)	27 (14,4)	----	------	1 (Reference)	1 (Reference)	------
II/A	6 (3,7)	11 (5,9)	0,10	0,748	1,43 (0,45–4,49)	0,98 (0,27–3,48)	0,970
ID/G	67 (41,6)	80 (42,8)	0,01	0,957	0,93 (0,48–1,79)	1,04 (0,51–2,12)	0,907
ID/A	19 (11,8)	20 (10,1)	0,06	0,806	0,82 (0,35–1,91)	0,99 (0,39–2,51)	0,979
DD/G	35 (21,7)	39 (20,8)	0,04	0,843	0,87 (0,42–1,80)	1,08 (0,50–2,37)	0,843
DD/A	13 (8,1)	10 (5,3)	0,57	0,451	0,60 (0,22–1,63)	0,70 (0,24–2,03)	0,506

Analysis by Chi-Square Test (χ^2^) or Fisher’s Exact Test (#), calculation of Odds Ratio (OR) with Confidence Interval (95%CI), and Multinomial Logistic Regression to obtain adjusted Odds Ratio (OR^A^). *p^1^* -p-value fchi-square test or Fisher’s exact test. *p^2^*—p-value for OR^A^. *Significant difference between groups (p<0.05). The notation A_ refers to AA and GA genotypes, while G_ refers to GG and GA genotypes of the ACE2 G8790A polymorphism.

The distribution of ACE and ACE2 genotypes (Table 5) for the sampled population was consistent with that expected by the Hardy-Weinberg Equilibrium, suggesting the absence of selective pressures involving the genetic *locus* evaluated and a representative sampling of the studied population in the study field.

**Table 5:** Hardy-Weinberg equilibrium test for ACE I/D and ACE2 G8790A polymorphisms in moderate and severe groups of COVID-19.

Genotype	Obs.	Exp.	χ^2^ (1 D. F.)	p	Alleles	Frequency
**ACE**						
Moderate Group						
II	58	56,2	0,177	0,6738	I	0,44
ID	142	145,6			D	0,56
DD	96	94,2				
Total	296	296				
Severe Group						
II	66	71,1	1,286	0,2568	I	0,47
ID	172	161,8			D	0,53
DD	87	92,1				
Total	325	325				
**ACE2**						
Moderade Group (Female)						
GG	68	65,5	1,075	0,2997	G	0,70
GA	52	57,1			A	0,30
AA	15	12,4				
Total	135	135				
Severe Group (Female)						
GG	81	79,9	0,2591	0,6040	G	0,76
GA	48	50,2			A	0,24
AA	9	7,9				
Total	138					

χ^2^ test Analysis. Obs–Observed; Exp–Expected; DF–Degree of Freedom. Significance level: p<0.05.

## Discussion

The possibility of a genetic predisposition to SARS-CoV-2 infection has been raised in the scientific literature, especially considering the role that polymorphisms of ACE and ACE2 could play in the infection process and disease progression, considering the activity of these enzymes in the pathophysiology of acute lung injury and in the invasion mechanism used by the new coronavirus [35–38]. The results of the present study point to the existence, already known in the literature, of health conditions as risk factors, as well as the unprecedented existence of a genetic protection factor regarding the susceptibility to worsening of the patient with COVID-19 mediated by the combined polymorphisms of *ACE* and *ACE2*.

As observed, the elderly (p<0.001) and the presence of comorbidities such as systemic arterial hypertension (p = 0.0310), diabetes *mellitus* (p = 0.0016), respiratory diseases (p = 0.0018), cardiovascular diseases (p = 0.0003), neuropsychiatric diseases and disorders (p = 0.0001) and acute and chronic kidney diseases (p = 0.0002) were significantly more frequent in individuals with more severe outcomes compared to less severe ones when infected with SARS-CoV-2, being associated with the worsening of the disease as independent risk factors. These associations are already well recognized in the literature for the Brazilian and worldwide populations [1, 39, 40], as previously expected, due to the systemic pathophysiological changes caused by COVID-19, especially in serious situations [41].

The study of the role played by key enzymes that make up RAS is essential for understanding the endogenous contributors to the worsening of COVID-19. The role of increased ACE2 expression in the complications of the disease is debated because that would facilitate virus entry into the cells, but at the same time, expressing less, or having less activity of this enzyme would also contribute to other aggravating conditions imposed by the virus that is involved in lung injury and multiorgan failure [3, 6, 7, 12, 42–44]. In this sense, the study of the impact of polymorphisms, epigenetic changes, and other molecular mechanisms in the modulation of ACE and ACE2 activity or expression is important to elucidate the worsening of the disease [16]. *ACE* I/D (rs2106809) and *ACE2* G8790A (rs2285666) polymorphisms are studied in a great variety of conditions by their effect on the plasma and tissue levels of these key RAS enzymes, having been described as functional polymorphisms [45]. It´s well known the ACE D allele relation with increased ACE activity [24, 25, 45], while evidence points to the ACE2 A allele association with enhanced gene expression and the possibility of interference in the protein product by alternative splicing [30, 46, 47]. The molecular mechanisms underlying these effects still need to be better elucidated.

In the results of this study, the genotypic profiles of *ACE* and *ACE2*, individually, did not represent susceptibility or protection for the patient affected by COVID-19. However, it was found that the genotypic combination of the II genotype of *ACE* with the A allele of *ACE2* (II/A_) was significantly more frequent in female individuals from the mild outcome group of COVID-19 (χ^2^ = 4.83; p = 0.028), presenting a protective role against the worsening of the disease (OR^A^ = 0.26; p = 0.037).

The association results obtained mean that II/A_ carriers have almost four times (1 over 0.26) less chance of a severe outcome in females. This result corroborates gender-related differences in COVID-19 susceptibility in the sense of a male increased mortality rate, leading to reflections concerning a possible differential ACE2 expression in woman involving mechanisms of X chromosome inactivation escape, epigenetic modifications, and sex hormones regulation [48]. Epigenetic modifications during aging, marked by reduced DNA methylation and reversion of inactivated genes, would also reflect changes in the incidence and prognosis of the disease [49].

This unprecedented result is in line with Hatami et al. 2020 [50], who have pointed to a possible association between the increase in I/D ratio and the increase in COVID-19 recovery rate by meta-regression analysis involving prevalence data from 30 different countries (point estimate: 0.48, CI 95%: 0.05–0.91, p  =  0.027). Then, the presence of the I allele and, mainly, the II genotype of ACE may be considered a benefical factor in disease progression.

In previous studies, the D allele or DD genotype of *ACE* has been associated with increased enzyme activity and implied susceptibility to diseases, such as hypertension and CVDs, in which there would be greater activity of the enzyme [20–22]. Thus, the I allele would be associated with lower ACE activity, resulting in lower Ang-II production, and, therefore, it would confer a more favorable physiological status to the success in coping with the pathology, leading to a lower pro-inflammatory cascade during the infection course, marked by ACE2 downregulation and, consequently, RAS imbalance.

In this line of evidence, Sieńko et al. (2020) [51], in a narrative review, pointed to the importance of the D allele in the risk of serious outcomes in COVID-19. Delanghe *et al*. (2020) [35] draw attention to an important geographical variation of *ACE*I/D polymorphism that began to be associated with COVID-19 prevalence and mortality data [36]. Then, an ecological study conducted by Yamamoto et al. (2020) [52] involving data from 26 countries in Europe and Asia reported a negative *ACE* II genotype correlation with the number of COVID-19 cases (R = − 0.847) and deaths (R = − 0.755).

Yamamoto et al. (2020) [52] highlight that ethnic-geographical influences can impact the findings of the studies, given that populations with different genetic backgrounds can have different outcomes. In this sense, the present work, despite not having found a profile of genetic susceptibility, is in line with the results indicated, once it was verified an association of protection for the II genotype of *ACE* when in combination with the A allele of *ACE2*, having to take into account that it sampled individuals from the Brazilian population, which is recognizably marked by great racial miscegenation in its origins and in its historical course.

In agreement with the findings of the present study, Manning & Fink (2020) [53] in an ecological study involving data on COVID-19 fatalities among 41 nations suggested that genotype II could have a protective role against worsening. The authors speak in favor of a supposed “oxygen mechanism”, according to which there would be a polymorphic contribution of the D allele to lung damage and reduced blood O2 saturation. In our study, it was not possible to verify an association of the D allele with aggravation in patients affected by the disease.

Similar results were verified by Aung et al. (2020) [54] in another ecological study contemplating 25 countries from diverse geographical regions of the world. The study pointed out that no association was found between the frequency of the DD genotype and COVID-19 mortality rates (OR = 4.3; p = 0.2). Despite this, the researchers verified that increased II genotype frequency was significantly associated with decreased COVID-19 mortality rates (IRR: 0.3; p = 0.03).

Regarding the G8790A polymorphism of the *ACE2* gene, in an ecological study involving Indian populations, Srivastava et al. (2020) [30] pointed to a correlation of the A allele with a lower rate of infection (r = −0.571; p = 0.021) and lethality (r = −0.699; p = 0.002) in patients affected by the disease. The result agrees with our work in the sense that a protective factor was observed for the A allele in patients already affected by COVID-19.

Otherwise, Möhlendick et al., (2021) [31] have identified the *ACE2* G allele and GG genotype as a genetic risk factor for SARS-CoV-2 infection and COVID-19 mortality, evaluating 297 SARS-CoV-2-positive and 253 SARS-CoV-2-negative tested patients in the German population. A lack of association for the *ACE* I/D polymorphism was observed. Although the cited association was not verified in our study, it corroborates the hypothesis of the functional aspects associated with *ACE2* polymorphisms, especially that the G allele is associated with greater pulmonary and systemic injuries due to the imbalance in the RAS with the decrease in ACE2 activity.

In this study, we did not aim at estimating the influence of genetic polymorphisms on the prevalence of infection by the disease, but rather at associating them with the type of clinical outcome during hospitalization. However, regarding the allelic frequencies, it can be observed that the frequencies obtained in this study are close to those found by Pinheiro et al. (2019) [22], evaluating 123 controls and 117 hypertensive patients in the Brazilian population, considering the control group, for both polymorphisms evaluated in severe and moderate groups, with *ACE* I/D frequencies around 0.5 for each allele and, for *ACE2*, a proportion about 0.2 for A and 0.8 for G alleles. Thus, it is likely that there would be no change in the rate of infection by COVID-19 associated with these polymorphisms for the Brazilian population.

The initial results of non-association for *ACE* and *ACE2* polymorphisms analyzed separately are in agreement with a case study conducted by Karakaş Çelik et al. (2021) [32] that evaluated 155 patients divided into three COVID-19 groups (mild, moderate, and severe); however, the referred research has not explored the genotypic combination analyses, which have brought interesting association findings to this work and have been an important tool to be employed on genetic association studies [22].

The synergistic effects of ACE and ACE2 are in accordance with their counterbalancing biological roles in RAS and constitute a finding that has already been highlighted in another study of our research group [22], which revealed an important association of the DD/G_ profile with hypertension susceptibility (OR^A^ = 3.57; p = 0.026), comorbidity, which is also a COVID-19 risk factor. Interestingly, the results obtained in this study indicate protection for the complementary combination profile (II/A_), which can be considered along the same line of understanding, since if we have susceptibility on one side, we can expect protection on the other.

Similarly, if in that study we have pointed out that the increased activity of ACE (DD genotype) in conjugation with decreased activity of ACE2 (G allele) could be the underlining mechanism in hypertension susceptibility, here we hypothesize that the associated opposite effects on enzyme activity in the complementary genotype profile, that is, decreased ACE activity in conjunction with the increased ACE2 activity in patients with the protective genotype (II/A_), should be the protective physiological mechanism involved in COVID-19 outcome.

It’s interesting to highlight epigenetic mechanisms, which include DNA methylation, histone post-translational modifications, and microRNAs, that can impact ACE2 expression, have been associated with conditions of disease [49]. When considering the findings of this study, the epigenetic inactivation of the A allele (protective) in heterozygous women (GA) would have a prejudicial effect, mainly on ACE DD individuals. Through aging, the reversion of epigenetic silencing in female would have a positive impact on heterozygotes due to the possibility of A allele reactivation. This would help explain aspects of the differential sex-related susceptibility to COVID-19, once male subjects are hemizygous for ACE2 gene, and, therefore, cannot be beneficed by these epigenetic changes.

Considering the survey of the current literature, attention is drawn to the scarcity of studies addressing the G8790A polymorphism of *ACE2* in the ontogeny of COVID-19 infection and entry of the virus into host cells, while most of the studies have been dedicated to exploring genetic variations in the coding region of the *ACE2* gene [55, 56] with important results being obtained regarding variants that interfere with the virus-receptor interaction [16, 55–57]. However, it is worth mentioning that polymorphisms that influence enzymatic activity, even in an intronic region, can bring interesting findings because they have the potential to impact the clinical course of COVID-19 by impairing RAS balance, as verified in this study and evidenced by the results of Khayat et al. (2020) [47].

So far, no other work has presented the results found in this study for the genetic association between *ACE* and *ACE2* polymorphisms. The non-association for each polymorphism alone supports the hypothesis of genetic complexity behind outcomes for COVID-19, which explains the difficulty in the search for completely effective treatments to combat severe outcomes for the disease. It is reasonable to assume that a greater understanding of polymorphisms associated with the worsening of COVID-19 is highly relevant in decision-making in public policies, whether, for example, in the development of vaccination strategies by genotypic risk groups, or in personalized care for each risk profile.

As limitations and complements to this study, we highlight the expansion of the sample, both in number and in population, as well as the evaluation of the concentration of detached circulating enzymes in the plasma. The corroboration of these results by further research can substantially benefit personalized medicine, which favors the adoption of measures linked to evidence-based medicine given the need to continue reducing mortality and controlling the disease.

## Conclusions

The study of genetic risk factors is one arm of personalized medicine development. The results obtained in this study indicate a protection profile for COVID-19 progression for the population under analysis, in which the II/A_ carriers have almost four times less chance to evolve to a severe outcome. This means that, not having this genetic combination can be a signal of poor prognosis for the patient.

The finding is unprecedented in the literature, as other studies have not explored the genotypic combination analysis for *ACE* and *ACE2* polymorphisms. In line with the literature, it is reasonable to assume that the protective alleles identified are associated with greater homeostasis in pulmonary RAS, reducing the pro-inflammatory profile generated by the disease, and thus preventing worse outcomes.

The number of independent risk factors, including genetic contributors, associated with aggravation or protection against COVID-19 identified in this and other research bring to light the multifactorial nature of the disease, with the study of new genes being highly suggestible for a better understanding of COVID-19 pathophysiology and the development of new treatment strategies.

## Supporting information

S1 File Data from mild and severe COVID-19 groups. (DOCX)
