# Supplementary material for: Combinatorial analysis of ACE and ACE2 polymorphisms reveals protection against COVID-19 worsening: A genetic association study in Brazilian patients
Source: PLoS One. 2023 Nov 30;18(11):e0288178. doi: 10.1371/journal.pone.0288178 (PMC10688632; doi:10.1371/journal.pone.0288178)
Supplement: S1 File — (DOCX) [file pone.0288178.s001.docx]

**S1 File.** Data from Mild and Severe COVID-19 groups

**MILD-COVID-19 GROUP**

| Patient Id | Age  (years) | Sex  (M=0  F=1) | Hypertension | | Diabetes | | RSDs | CVD | Obesity | NDDs | ACKDs | Smoking | | ACE ID  (II=1; ID=2; DD=3) | ACE2 G8790A  F: GG=1, GA=2, AA=3  M: G=1; A=3 |
| --- | --- | --- | --- | --- | --- | --- | --- | --- | --- | --- | --- | --- | --- | --- | --- |
| MILD-103 | 42 | 1 | 0 | 0 | | 0 | | 0 | 0 | 0 | 0 | | 0 | 3 | 1 |
| MILD-104 | 42 | 1 | 0 | 0 | | 0 | | 0 | 0 | 0 | 0 | | 0 | 2 | 3 |
| MILD-108 | 73 | 0 | 1 | 0 | | 0 | | 0 | 0 | 0 | 0 | | 0 | 1 | 1 |
| MILD-112 | 36 | 1 | 0 | 0 | | 0 | | 0 | 0 | 0 | 0 | | 0 | 2 | 1 |
| MILD-117 | 68 | 0 | 1 | 1 | | 0 | | 0 | 0 | 0 | 0 | | 0 | 2 | 1 |
| MILD-120 | 39 | 0 | 1 | 0 | | 0 | | 0 | 0 | 0 | 0 | | 0 | 3 | 1 |
| MILD-123 | 35 | 1 | 0 | 0 | | 0 | | 0 | 0 | 0 | 0 | | 0 | 3 | 2 |
| MILD-132 | 33 | 1 | 0 | 0 | | 0 | | 0 | 0 | 0 | 0 | | 0 | 1 | 1 |
| MILD-133 | 61 | 1 | 1 | 0 | | 0 | | 0 | 0 | 0 | 0 | | 0 | 3 | 1 |
| MILD-142 | 54 | 0 | 1 | 0 | | 0 | | 1 | 0 | 0 | 0 | | 0 | 2 | 1 |
| MILD-156 | 52 | 1 | 1 | 0 | | 0 | | 0 | 0 | 0 | 0 | | 0 | 2 | 1 |
| MILD-157 | 60 | 0 | 1 | 1 | | 0 | | 0 | 0 | 0 | 0 | | 0 | 1 | 1 |
| MILD-159 | 44 | 1 | 0 | 0 | | 0 | | 0 | 0 | 0 | 0 | | 0 | 2 | 1 |
| MILD-160 | 60 | 0 | 0 | 0 | | 0 | | 0 | 0 | 0 | 0 | | 0 | 3 | 1 |
| MILD-162 | 56 | 1 | 0 | 0 | | 0 | | 0 | 0 | 0 | 0 | | 0 | 3 | 1 |
| MILD-164 | 57 | 1 | 0 | 0 | | 0 | | 0 | 0 | 0 | 0 | | 0 | 3 | 1 |
| MILD-165 | 56 | 1 | 0 | 0 | | 0 | | 0 | 0 | 0 | 0 | | 0 | 1 | 3 |
| MILD-168 | 56 | 0 | 0 | 0 | | 0 | | 0 | 0 | 0 | 0 | | 0 | 2 | 1 |
| MILD-172 | 59 | 1 | 0 | 0 | | 0 | | 0 | 0 | 0 | 0 | | 0 | 2 | 2 |
| MILD-173 | 43 | 1 | 0 | 0 | | 0 | | 0 | 0 | 0 | 0 | | 0 | 3 | 1 |
| MILD-175 | 92 | 1 | 1 | 0 | | 0 | | 0 | 0 | 0 | 0 | | 0 | 3 | 3 |
| MILD-176 | 80 | 1 | 0 | 0 | | 0 | | 1 | 0 | 0 | 0 | | 0 | 2 | 2 |
| MILD-177 | 61 | 0 | 0 | 0 | | 0 | | 0 | 0 | 0 | 0 | | 0 | 2 | 1 |
| MILD-178 | 79 | 1 | 1 | 1 | | 0 | | 0 | 0 | 0 | 0 | | 0 | 3 | 1 |
| MILD-183 | 48 | 1 | 0 | 0 | | 0 | | 0 | 0 | 0 | 0 | | 0 | 1 | 2 |
| MILD-184 | 65 | 0 | 0 | 0 | | 0 | | 0 | 0 | 0 | 0 | | 0 | 3 | 3 |
| MILD-185 | 71 | 1 | 1 | 1 | | 0 | | 1 | 0 | 0 | 0 | | 0 | 3 | 1 |
| MILD-186 | 45 | 0 | 1 | 1 | | 0 | | 1 | 0 | 0 | 0 | | 0 | 3 | 1 |
| MILD-188 | 48 | 1 | 0 | 0 | | 0 | | 0 | 0 | 1 | 0 | | 0 | 2 | 1 |
| MILD-189 | 79 | 0 | 0 | 0 | | 0 | | 0 | 0 | 0 | 0 | | 0 | 3 | 1 |
| MILD-190 | 35 | 1 | 1 | 0 | | 0 | | 0 | 1 | 0 | 0 | | 0 | 2 | 1 |
| MILD-192 | 31 | 1 | 0 | 0 | | 0 | | 0 | 0 | 0 | 0 | | 0 | 1 | 1 |
| MILD-196 | 73 | 0 | 0 | 0 | | 0 | | 0 | 0 | 0 | 0 | | 0 | 1 | 1 |
| MILD-197 | 51 | 0 | 0 | 0 | | 0 | | 0 | 0 | 0 | 0 | | 0 | 3 | 3 |
| MILD-198 | 69 | 0 | 0 | 0 | | 0 | | 0 | 0 | 0 | 0 | | 0 | 3 | 3 |
| MILD-201 | 54 | 1 | 1 | 0 | | 0 | | 0 | 0 | 0 | 0 | | 0 | 3 | 1 |
| MILD-202 | 31 | 0 | 0 | 0 | | 0 | | 0 | 0 | 0 | 0 | | 0 | 2 | 3 |
| MILD-203 | 62 | 1 | 1 | 0 | | 0 | | 0 | 0 | 0 | 0 | | 0 | 2 | 2 |
| MILD-206 | 51 | 1 | 0 | 0 | | 0 | | 0 | 0 | 0 | 0 | | 0 | 1 | 2 |
| MILD-207 | 66 | 0 | 0 | 0 | | 0 | | 0 | 0 | 0 | 0 | | 0 | 2 | 1 |
| MILD-208 | 53 | 0 | 0 | 0 | | 0 | | 0 | 0 | 0 | 0 | | 0 | 2 | 1 |
| MILD-209 | 65 | 1 | 0 | 0 | | 0 | | 0 | 0 | 0 | 0 | | 0 | 3 | 2 |
| MILD-212 | 92 | 0 | 0 | 0 | | 0 | | 0 | 0 | 0 | 0 | | 0 | 1 | 1 |
| MILD-213 | 26 | 0 | 0 | 0 | | 0 | | 0 | 0 | 0 | 0 | | 0 | 2 | 1 |
| MILD-214 | 71 | 0 | 0 | 0 | | 0 | | 0 | 0 | 0 | 0 | | 0 | 2 | 3 |
| MILD-215 | 55 | 0 | 0 | 0 | | 0 | | 0 | 0 | 0 | 0 | | 0 | 3 | 1 |
| MILD-216 | 40 | 1 | 0 | 0 | | 0 | | 0 | 0 | 0 | 0 | | 0 | 2 | 2 |
| MILD-218 | 42 | 0 | 0 | 0 | | 0 | | 0 | 0 | 0 | 0 | | 0 | 2 | 3 |
| MILD-219 | 58 | 1 | 0 | 0 | | 0 | | 0 | 0 | 0 | 0 | | 0 | 3 | 1 |
| MILD-220 | 51 | 1 | 0 | 0 | | 0 | | 0 | 0 | 0 | 0 | | 0 | 2 | 2 |
| MILD-221 | 89 | 1 | 0 | 0 | | 0 | | 0 | 0 | 0 | 0 | | 0 | 2 | 1 |
| MILD-222 | 46 | 1 | 0 | 0 | | 0 | | 0 | 0 | 0 | 0 | | 0 | 2 | 2 |
| MILD-223 | 55 | 0 | 0 | 0 | | 1 | | 0 | 1 | 0 | 0 | | 0 | 3 | 1 |
| MILD-226 | 36 | 0 | 0 | 0 | | 0 | | 0 | 0 | 0 | 0 | | 0 | 3 | 1 |
| MILD-228 | 56 | 1 | 0 | 0 | | 0 | | 0 | 0 | 0 | 0 | | 0 | 3 | 1 |
| MILD-229 | 58 | 0 | 1 | 1 | | 0 | | 0 | 0 | 0 | 0 | | 0 | 1 | 1 |
| MILD-236 | 73 | 1 | 0 | 0 | | 0 | | 0 | 0 | 0 | 0 | | 0 | 2 | 2 |
| MILD-240 | 48 | 1 | 0 | 0 | | 0 | | 0 | 0 | 0 | 0 | | 0 | 1 | 1 |
| MILD-242 | 46 | 1 | 0 | 0 | | 0 | | 0 | 0 | 0 | 0 | | 0 | 3 | 1 |
| MILD-244 | 52 | 0 | 0 | 0 | | 0 | | 0 | 0 | 0 | 0 | | 0 | 1 | 1 |
| MILD-245 | 51 | 1 | 0 | 0 | | 1 | | 0 | 0 | 0 | 0 | | 1 | 3 | 1 |
| MILD-247 | 47 | 0 | 1 | 1 | | 1 | | 0 | 0 | 0 | 0 | | 1 | 2 | 1 |
| MILD-250 | 64 | 0 | 0 | 0 | | 0 | | 0 | 0 | 0 | 0 | | 0 | 3 | 1 |
| MILD-251 | 45 | 0 | 0 | 0 | | 0 | | 0 | 0 | 0 | 0 | | 0 | 2 | 1 |
| MILD-252 | 23 | 1 | 0 | 0 | | 0 | | 0 | 0 | 0 | 0 | | 0 | 2 | 1 |
| MILD-254 | 67 | 0 | 1 | 1 | | 0 | | 1 | 0 | 0 | 0 | | 0 | 2 | 1 |
| MILD-255 | 49 | 0 | 0 | 0 | | 0 | | 0 | 0 | 0 | 0 | | 0 | 3 | 1 |
| MILD-257 | 35 | 0 | 1 | 1 | | 0 | | 0 | 0 | 0 | 0 | | 0 | 3 | 1 |
| MILD-261 | 60 | 0 | 1 | 0 | | 0 | | 0 | 0 | 0 | 0 | | 0 | 3 | 1 |
| MILD-264 | 55 | 1 | 0 | 0 | | 0 | | 0 | 0 | 0 | 0 | | 0 | 2 | 2 |
| MILD-267 | 21 | 1 | 0 | 0 | | 0 | | 0 | 0 | 0 | 0 | | 0 | 2 | 2 |
| MILD-271 | 39 | 1 | 1 | 0 | | 0 | | 0 | 0 | 0 | 0 | | 0 | 2 | 2 |
| MILD-272 | 56 | 0 | 0 | 0 | | 0 | | 0 | 0 | 0 | 0 | | 0 | 2 | 3 |
| MILD-273 | 28 | 1 | 0 | 0 | | 1 | | 0 | 0 | 1 | 0 | | 0 | 2 | 3 |
| MILD-274 | 42 | 1 | 0 | 0 | | 0 | | 0 | 0 | 0 | 0 | | 0 | 3 | 3 |
| MILD-275 | 46 | 1 | 0 | 0 | | 0 | | 0 | 0 | 0 | 0 | | 0 | 3 | 2 |
| MILD-278 | 51 | 0 | 0 | 0 | | 0 | | 0 | 0 | 0 | 0 | | 0 | 3 | 1 |
| MILD-280 | 72 | 0 | 0 | 0 | | 1 | | 0 | 0 | 0 | 0 | | 1 | 2 | 3 |
| MILD-283 | 77 | 0 | 0 | 0 | | 0 | | 0 | 0 | 0 | 0 | | 0 | 3 | 3 |
| MILD-285 | 83 | 0 | 0 | 0 | | 0 | | 0 | 0 | 0 | 0 | | 0 | 2 | 1 |
| MILD-288 | 61 | 1 | 0 | 0 | | 0 | | 0 | 0 | 0 | 0 | | 0 | 2 | 2 |
| MILD-291 | 85 | 1 | 0 | 0 | | 0 | | 0 | 0 | 0 | 0 | | 0 | 2 | 3 |
| MILD-293 | 41 | 1 | 1 | 0 | | 0 | | 0 | 0 | 0 | 0 | | 0 | 3 | 3 |
| MILD-294 | 69 | 0 | 0 | 0 | | 0 | | 0 | 0 | 0 | 0 | | 0 | 2 | 1 |
| MILD-297 | 65 | 1 | 1 | 1 | | 0 | | 0 | 0 | 1 | 0 | | 0 | 2 | 2 |
| MILD-302 | 72 | 0 | 0 | 0 | | 0 | | 0 | 0 | 0 | 0 | | 0 | 2 | 1 |
| MILD-305 | 55 | 0 | 1 | 0 | | 0 | | 0 | 1 | 0 | 0 | | 0 | 3 | 1 |
| MILD-312 | 71 | 1 | 0 | 0 | | 0 | | 0 | 0 | 0 | 0 | | 0 | 3 | 2 |
| MILD-314 | 56 | 0 | 0 | 0 | | 0 | | 0 | 0 | 0 | 0 | | 0 | 2 | 3 |
| MILD-316 | 75 | 1 | 0 | 0 | | 0 | | 0 | 0 | 0 | 0 | | 0 | 1 | 2 |
| MILD-322 | 51 | 1 | 0 | 0 | | 0 | | 0 | 0 | 0 | 0 | | 0 | 2 | 1 |
| MILD-324 | 81 | 0 | 1 | 1 | | 0 | | 0 | 0 | 0 | 0 | | 0 | 2 | 3 |
| MILD-325 | 52 | 0 | 0 | 0 | | 0 | | 0 | 0 | 0 | 0 | | 0 | 2 | 1 |
| MILD-326 | 64 | 0 | 0 | 0 | | 0 | | 0 | 0 | 0 | 0 | | 0 | 2 | 1 |
| MILD-328 | 88 | 0 | 0 | 0 | | 0 | | 1 | 0 | 0 | 0 | | 0 | 2 | 1 |
| MILD-330 | 78 | 1 | 0 | 0 | | 0 | | 0 | 0 | 0 | 0 | | 0 | 2 | 2 |
| MILD-331 | 48 | 0 | 0 | 0 | | 0 | | 0 | 0 | 0 | 0 | | 0 | 2 | 1 |
| MILD-332 | 79 | 1 | 1 | 0 | | 0 | | 0 | 0 | 0 | 0 | | 0 | 3 | 1 |
| MILD-359 | 35 | 0 | 1 | 0 | | 0 | | 0 | 0 | 0 | 0 | | 0 | 2 | 1 |
| MILD-361 | 67 | 0 | 0 | 0 | | 0 | | 0 | 0 | 0 | 0 | | 0 | 3 | 1 |
| MILD-362 | 41 | 1 | 0 | 0 | | 0 | | 0 | 0 | 0 | 0 | | 0 | 1 | 2 |
| MILD-363 | 48 | 0 | 0 | 0 | | 0 | | 0 | 0 | 0 | 0 | | 0 | 3 | 3 |
| MILD-364 | 42 | 0 | 0 | 0 | | 0 | | 0 | 0 | 0 | 0 | | 0 | 3 | 1 |
| MILD-365 | 47 | 0 | 0 | 0 | | 0 | | 0 | 0 | 0 | 0 | | 0 | 1 | 1 |
| MILD-367 | 36 | 0 | 0 | 0 | | 0 | | 0 | 0 | 0 | 0 | | 0 | 2 | 3 |
| MILD-369 | 55 | 0 | 1 | 0 | | 0 | | 0 | 0 | 0 | 0 | | 0 | 2 | 1 |
| MILD-371 | 64 | 0 | 0 | 0 | | 1 | | 0 | 0 | 0 | 0 | | 1 | 2 | 1 |
| MILD-374 | 31 | 0 | 0 | 0 | | 0 | | 0 | 0 | 0 | 0 | | 0 | 2 | 3 |
| MILD-377 | 63 | 1 | 0 | 0 | | 0 | | 0 | 0 | 0 | 0 | | 0 | 2 | 2 |
| MILD-379 | 54 | 0 | 0 | 0 | | 0 | | 0 | 0 | 0 | 0 | | 0 | 1 | 3 |
| MILD-380 | 72 | 1 | 1 | 0 | | 0 | | 0 | 0 | 0 | 0 | | 0 | 3 | 1 |
| MILD-382 | 66 | 0 | 0 | 0 | | 0 | | 0 | 0 | 0 | 0 | | 0 | 2 | 3 |
| MILD-385 | 38 | 0 | 0 | 0 | | 0 | | 0 | 0 | 0 | 0 | | 0 | 3 | 1 |
| MILD-386 | 56 | 0 | 0 | 0 | | 0 | | 0 | 0 | 0 | 0 | | 0 | 3 | 1 |
| MILD-388 | 23 | 0 | 0 | 0 | | 0 | | 0 | 0 | 0 | 0 | | 0 | 3 | 1 |
| MILD-389 | 73 | 0 | 1 | 1 | | 0 | | 0 | 1 | 0 | 0 | | 0 | 3 | 1 |
| MILD-391 | 47 | 0 | 0 | 0 | | 0 | | 0 | 0 | 0 | 0 | | 0 | 2 | 1 |
| MILD-395 | 85 | 1 | 1 | 0 | | 0 | | 0 | 0 | 0 | 0 | | 0 | 3 | 1 |
| MILD-396 | 38 | 1 | 0 | 0 | | 0 | | 0 | 0 | 0 | 0 | | 0 | 1 | 1 |
| MILD-397 | 55 | 1 | 0 | 0 | | 0 | | 0 | 0 | 0 | 0 | | 0 | 3 | 1 |
| MILD-403 | 66 | 1 | 0 | 0 | | 0 | | 0 | 0 | 0 | 0 | | 0 | 1 | 1 |
| MILD-405 | 65 | 0 | 0 | 0 | | 0 | | 0 | 0 | 0 | 0 | | 0 | 3 | 1 |
| MILD-407 | 77 | 0 | 1 | 0 | | 0 | | 0 | 0 | 0 | 0 | | 0 | 3 | 1 |
| MILD-409 | 62 | 0 | 0 | 0 | | 0 | | 0 | 0 | 0 | 0 | | 0 | 2 | 1 |
| MILD-410 | 69 | 0 | 1 | 1 | | 0 | | 0 | 0 | 0 | 0 | | 0 | 3 | 1 |
| MILD-412 | 47 | 0 | 0 | 0 | | 0 | | 0 | 0 | 0 | 0 | | 0 | 3 | 1 |
| MILD-413 | 66 | 0 | 1 | 0 | | 0 | | 0 | 0 | 0 | 0 | | 0 | 2 | 1 |
| MILD-415 | 76 | 0 | 0 | 0 | | 0 | | 0 | 0 | 0 | 0 | | 0 | 2 | 1 |
| MILD-417 | 59 | 0 | 1 | 1 | | 0 | | 1 | 0 | 0 | 0 | | 0 | 1 | 1 |
| MILD-418 | 82 | 1 | 1 | 0 | | 0 | | 0 | 0 | 0 | 0 | | 0 | 2 | 1 |
| MILD-419 | 68 | 1 | 0 | 0 | | 0 | | 0 | 0 | 0 | 0 | | 0 | 3 | 2 |
| MILD-420 | 53 | 0 | 0 | 0 | | 0 | | 0 | 0 | 0 | 0 | | 0 | 2 | 1 |
| MILD-422 | 64 | 0 | 0 | 0 | | 0 | | 0 | 0 | 0 | 0 | | 0 | 2 | 1 |
| MILD-423 | 71 | 0 | 0 | 0 | | 0 | | 0 | 0 | 0 | 0 | | 0 | 2 | 1 |
| MILD-429 | 67 | 1 | 0 | 0 | | 0 | | 0 | 0 | 0 | 0 | | 0 | 2 | 2 |
| MILD-430 | 68 | 1 | 0 | 0 | | 0 | | 0 | 0 | 0 | 0 | | 0 | 2 | 1 |
| MILD-433 | 57 | 1 | 0 | 0 | | 0 | | 0 | 0 | 0 | 0 | | 0 | 1 | 1 |
| MILD-436 | 88 | 0 | 1 | 0 | | 0 | | 0 | 0 | 0 | 0 | | 0 | 3 | 1 |
| MILD-437 | 68 | 1 | 1 | 0 | | 0 | | 0 | 0 | 0 | 0 | | 0 | 3 | 2 |
| MILD-440 | 77 | 0 | 0 | 0 | | 0 | | 0 | 0 | 0 | 0 | | 0 | 3 | 1 |
| MILD-442 | 71 | 1 | 0 | 0 | | 0 | | 0 | 0 | 0 | 0 | | 0 | 2 | 3 |
| MILD-443 | 74 | 0 | 0 | 0 | | 0 | | 0 | 0 | 0 | 0 | | 0 | 2 | 1 |
| MILD-444 | 62 | 1 | 0 | 0 | | 0 | | 0 | 0 | 0 | 0 | | 0 | 3 | 1 |
| MILD-445 | 46 | 0 | 0 | 0 | | 0 | | 0 | 0 | 1 | 0 | | 0 | 2 | 1 |
| MILD-450 | 32 | 1 | 0 | 0 | | 0 | | 0 | 0 | 0 | 0 | | 0 | 1 | 2 |
| MILD-454 | 61 | 1 | 1 | 1 | | 1 | | 0 | 0 | 0 | 0 | | 0 | 3 | 2 |
| MILD-456 | 38 | 0 | 0 | 0 | | 0 | | 0 | 0 | 0 | 0 | | 0 | 2 | 1 |
| MILD-457 | 38 | 1 | 0 | 0 | | 0 | | 0 | 0 | 0 | 0 | | 0 | 1 | 1 |
| MILD-458 | 80 | 1 | 0 | 0 | | 0 | | 0 | 0 | 0 | 0 | | 0 | 1 | 1 |
| MILD-460 | 46 | 0 | 0 | 0 | | 0 | | 0 | 0 | 0 | 0 | | 0 | 2 | 1 |
| MILD-464 | 83 | 1 | 0 | 0 | | 0 | | 0 | 0 | 0 | 0 | | 0 | 1 | 2 |
| MILD-466 | 79 | 1 | 1 | 0 | | 0 | | 1 | 0 | 0 | 0 | | 0 | 1 | 1 |
| MILD-468 | 73 | 0 | 0 | 1 | | 1 | | 0 | 0 | 0 | 0 | | 1 | 1 | 3 |
| MILD-473 | 59 | 0 | 0 | 1 | | 0 | | 1 | 0 | 0 | 0 | | 0 | 2 | 1 |
| MILD-478 | 60 | 0 | 0 | 0 | | 1 | | 0 | 0 | 0 | 0 | | 0 | 2 | 1 |
| MILD-495 | 74 | 1 | 1 | 0 | | 0 | | 1 | 0 | 0 | 0 | | 0 | 1 | 2 |
| MILD-496 | 76 | 0 | 0 | 0 | | 0 | | 0 | 0 | 0 | 0 | | 0 | 2 | 3 |
| MILD-498 | 68 | 0 | 0 | 0 | | 0 | | 1 | 0 | 0 | 0 | | 0 | 1 | 1 |
| MILD-500 | 51 | 0 | 0 | 0 | | 0 | | 0 | 0 | 0 | 0 | | 0 | 2 | 1 |
| MILD-504 | 61 | 0 | 1 | 0 | | 0 | | 0 | 0 | 0 | 0 | | 0 | 1 | 1 |
| MILD-507 | 74 | 1 | 1 | 1 | | 0 | | 0 | 0 | 1 | 0 | | 0 | 1 | 3 |
| MILD-511 | 55 | 1 | 1 | 1 | | 0 | | 0 | 1 | 0 | 0 | | 0 | 2 | 1 |
| MILD-520 | 68 | 1 | 0 | 0 | | 0 | | 0 | 0 | 0 | 0 | | 0 | 3 | 1 |
| MILD-537 | 59 | 0 | 0 | 0 | | 1 | | 1 | 0 | 0 | 0 | | 1 | 3 | 1 |
| MILD-539 | 70 | 0 | 1 | 1 | | 1 | | 0 | 0 | 0 | 0 | | 0 | 2 | 1 |
| MILD-540 | 76 | 0 | 0 | 1 | | 0 | | 0 | 0 | 0 | 0 | | 0 | 2 | 1 |
| MILD-541 | 38 | 1 | 0 | 0 | | 1 | | 0 | 0 | 0 | 0 | | 1 | 2 | 3 |
| MILD-545 | 35 | 0 | 0 | 0 | | 0 | | 0 | 0 | 0 | 0 | | 0 | 3 | 3 |
| MILD-546 | 69 | 1 | 0 | 0 | | 0 | | 0 | 0 | 0 | 0 | | 0 | 3 | 1 |
| MILD-549 | 39 | 1 | 1 | 1 | | 0 | | 0 | 0 | 0 | 0 | | 0 | 1 | 2 |
| MILD-551 | 37 | 1 | 0 | 0 | | 0 | | 0 | 1 | 0 | 0 | | 0 | 3 | 1 |
| MILD-560 | 59 | 1 | 0 | 0 | | 0 | | 0 | 0 | 0 | 0 | | 0 | 2 | 2 |
| MILD-561 | 77 | 0 | 1 | 0 | | 1 | | 1 | 0 | 1 | 0 | | 1 | 2 | 1 |
| MILD-564 | 49 | 0 | 1 | 0 | | 0 | | 0 | 1 | 0 | 0 | | 0 | 2 | 1 |
| MILD-566 | 85 | 1 | 1 | 0 | | 0 | | 0 | 0 | 1 | 0 | | 0 | 1 | 3 |
| MILD-575 | 49 | 0 | 0 | 0 | | 0 | | 0 | 0 | 0 | 0 | | 0 | 3 | 1 |
| MILD-576 | 50 | 1 | 0 | 0 | | 0 | | 0 | 0 | 0 | 0 | | 0 | 3 | 2 |
| MILD-577 | 60 | 0 | 0 | 0 | | 0 | | 0 | 0 | 0 | 0 | | 0 | 2 | 1 |
| MILD-578 | 48 | 0 | 0 | 0 | | 0 | | 0 | 0 | 0 | 0 | | 0 | 2 | 1 |
| MILD-580 | 80 | 0 | 1 | 0 | | 0 | | 0 | 0 | 0 | 0 | | 0 | 1 | 1 |
| MILD-582 | 52 | 0 | 1 | 0 | | 0 | | 0 | 0 | 0 | 0 | | 0 | 2 | 1 |
| MILD-586 | 53 | 0 | 0 | 0 | | 0 | | 0 | 0 | 0 | 0 | | 0 | 2 | 1 |
| MILD-589 | 28 | 1 | 0 | 0 | | 0 | | 0 | 0 | 0 | 0 | | 0 | 3 | 1 |
| MILD-590 | 53 | 0 | 0 | 0 | | 0 | | 0 | 0 | 0 | 0 | | 0 | 3 | 1 |
| MILD-598 | 62 | 0 | 1 | 1 | | 0 | | 0 | 0 | 0 | 0 | | 0 | 3 | 1 |
| MILD-600 | 45 | 0 | 0 | 0 | | 0 | | 0 | 0 | 0 | 0 | | 0 | 3 | 3 |
| MILD-605 | 89 | 1 | 1 | 1 | | 0 | | 0 | 0 | 0 | 0 | | 0 | 2 | 1 |
| MILD-609 | 62 | 0 | 1 | 1 | | 1 | | 0 | 0 | 0 | 0 | | 1 | 2 | 1 |
| MILD-615 | 76 | 0 | 1 | 0 | | 1 | | 0 | 0 | 0 | 0 | | 1 | 3 | 1 |
| MILD-617 | 50 | 0 | 0 | 0 | | 0 | | 0 | 0 | 0 | 0 | | 0 | 2 | 1 |
| MILD-618 | 68 | 0 | 1 | 0 | | 0 | | 0 | 0 | 0 | 0 | | 0 | 1 | 1 |
| MILD-619 | 44 | 1 | 0 | 0 | | 0 | | 0 | 0 | 0 | 0 | | 0 | 3 | 1 |
| MILD-621 | 83 | 0 | 1 | 1 | | 0 | | 0 | 0 | 0 | 0 | | 0 | 3 | 3 |
| MILD-623 | 56 | 0 | 1 | 1 | | 0 | | 0 | 0 | 0 | 0 | | 0 | 1 | 1 |
| MILD-626 | 72 | 1 | 1 | 1 | | 0 | | 0 | 0 | 0 | 0 | | 0 | 2 | 1 |
| MILD-627 | 41 | 1 | 0 | 0 | | 0 | | 0 | 0 | 0 | 0 | | 0 | 3 | 1 |
| MILD-628 | 57 | 1 | 0 | 0 | | 0 | | 0 | 0 | 0 | 0 | | 0 | 2 | 2 |
| MILD-629 | 34 | 0 | 0 | 0 | | 0 | | 0 | 0 | 0 | 0 | | 0 | 2 | 3 |
| MILD-633 | 15 | 0 | 0 | 0 | | 1 | | 0 | 0 | 0 | 0 | | 0 | 1 | 1 |
| MILD-634 | 52 | 0 | 1 | 0 | | 0 | | 0 | 0 | 0 | 0 | | 0 | 1 | 1 |
| MILD-635 | 71 | 1 | 1 | 1 | | 0 | | 0 | 0 | 0 | 0 | | 0 | 2 | 2 |
| MILD-639 | 49 | 0 | 0 | 0 | | 0 | | 0 | 0 | 0 | 0 | | 0 | 2 | 3 |
| MILD-640 | 39 | 1 | 0 | 0 | | 0 | | 0 | 0 | 0 | 0 | | 0 | 2 | 2 |
| MILD-648 | 78 | 1 | 0 | 0 | | 0 | | 0 | 0 | 1 | 0 | | 0 | 2 | 2 |
| MILD-650 | 41 | 1 | 0 | 0 | | 1 | | 0 | 0 | 1 | 1 | | 0 | 1 | 1 |
| MILD-651 | 46 | 0 | 0 | 1 | | 0 | | 0 | 0 | 0 | 0 | | 0 | 2 | 1 |
| MILD-653 | 78 | 0 | 0 | 0 | | 0 | | 0 | 0 | 0 | 0 | | 0 | 2 | 1 |
| MILD-658 | 57 | 0 | 0 | 0 | | 1 | | 0 | 1 | 0 | 0 | | 1 | 2 | 1 |
| MILD-660 | 65 | 1 | 0 | 0 | | 0 | | 0 | 0 | 0 | 0 | | 0 | 1 | 1 |
| MILD-664 | 70 | 1 | 1 | 0 | | 0 | | 0 | 0 | 0 | 0 | | 0 | 3 | 2 |
| MILD-667 | 54 | 0 | 0 | 0 | | 0 | | 0 | 0 | 0 | 0 | | 0 | 2 | 3 |
| MILD-668 | 45 | 1 | 1 | 1 | | 0 | | 0 | 0 | 1 | 0 | | 0 | 3 | 2 |
| MILD-675 | 67 | 0 | 1 | 1 | | 0 | | 0 | 0 | 0 | 0 | | 0 | 1 | 1 |
| MILD-678 | 38 | 0 | 1 | 0 | | 1 | | 0 | 1 | 0 | 0 | | 1 | 3 | 1 |
| MILD-679 | 49 | 1 | 0 | 0 | | 0 | | 0 | 0 | 1 | 0 | | 0 | 2 | 2 |
| MILD-682 | 66 | 0 | 0 | 0 | | 0 | | 0 | 0 | 0 | 0 | | 0 | 2 | 1 |
| MILD-687 | 57 | 0 | 1 | 1 | | 0 | | 0 | 0 | 0 | 0 | | 0 | 2 | 1 |
| MILD-688 | 59 | 0 | 0 | 0 | | 0 | | 0 | 0 | 0 | 0 | | 0 | 1 | 3 |
| MILD-692 | 44 | 0 | 0 | 0 | | 0 | | 0 | 1 | 0 | 0 | | 0 | 1 | 1 |
| MILD-695 | 65 | 1 | 1 | 1 | | 0 | | 0 | 0 | 0 | 0 | | 0 | 1 | 1 |
| MILD-700 | 57 | 0 | 1 | 0 | | 0 | | 0 | 0 | 0 | 0 | | 0 | 3 | 3 |
| MILD-701 | 45 | 1 | 0 | 0 | | 0 | | 0 | 0 | 1 | 0 | | 0 | 2 | 1 |
| MILD-707 | 51 | 0 | 0 | 0 | | 0 | | 0 | 0 | 0 | 0 | | 0 | 1 | 1 |
| MILD-708 | 70 | 1 | 1 | 1 | | 1 | | 0 | 0 | 0 | 0 | | 1 | 2 | 2 |
| MILD-713 | 68 | 0 | 1 | 0 | | 0 | | 0 | 0 | 1 | 0 | | 0 | 2 | 1 |
| MILD-716 | 52 | 1 | 0 | 0 | | 0 | | 0 | 0 | 0 | 0 | | 0 | 3 | 1 |
| MILD-717 | 77 | 0 | 1 | 0 | | 0 | | 1 | 0 | 0 | 0 | | 0 | 2 | 3 |
| MILD-722 | 66 | 0 | 1 | 0 | | 0 | | 0 | 0 | 0 | 0 | | 0 | 2 | 1 |
| MILD-727 | 30 | 1 | 0 | 0 | | 0 | | 0 | 0 | 0 | 0 | | 0 | 3 | 1 |
| MILD-732 | 85 | 0 | 1 | 1 | | 0 | | 0 | 0 | 0 | 0 | | 0 | 1 | 1 |
| MILD-733 | 69 | 0 | 1 | 1 | | 1 | | 1 | 0 | 0 | 0 | | 1 | 2 | 1 |
| MILD-734 | 59 | 1 | 1 | 0 | | 0 | | 0 | 0 | 1 | 0 | | 0 | 3 | 2 |
| MILD-735 | 62 | 1 | 1 | 0 | | 0 | | 0 | 0 | 0 | 0 | | 0 | 1 | 1 |
| MILD-736 | 62 | 0 | 0 | 0 | | 0 | | 0 | 0 | 0 | 0 | | 0 | 2 | 3 |
| MILD-740 | 72 | 0 | 0 | 0 | | 0 | | 0 | 0 | 0 | 0 | | 0 | 2 | 1 |
| MILD-741 | 57 | 0 | 0 | 0 | | 0 | | 0 | 0 | 0 | 0 | | 0 | 3 | 1 |
| MILD-744 | 67 | 1 | 1 | 1 | | 0 | | 0 | 0 | 0 | 0 | | 0 | 2 | 3 |
| MILD-745 | 29 | 0 | 0 | 0 | | 0 | | 0 | 0 | 0 | 0 | | 0 | 2 | 1 |
| MILD-747 | 37 | 1 | 1 | 0 | | 0 | | 0 | 0 | 0 | 0 | | 0 | 3 | 2 |
| MILD-749 | 57 | 1 | 1 | 0 | | 0 | | 0 | 0 | 0 | 0 | | 0 | 1 | 1 |
| MILD-755 | 48 | 1 | 0 | 0 | | 0 | | 0 | 0 | 0 | 0 | | 0 | 2 | 1 |
| MILD-757 | 40 | 0 | 1 | 0 | | 0 | | 0 | 0 | 0 | 0 | | 0 | 2 | 1 |
| MILD-758 | 68 | 0 | 0 | 0 | | 0 | | 0 | 0 | 0 | 0 | | 0 | 1 | 3 |
| MILD-759 | 70 | 0 | 0 | 0 | | 0 | | 0 | 0 | 0 | 0 | | 0 | 3 | 1 |
| MILD-760 | 70 | 1 | 1 | 0 | | 0 | | 0 | 0 | 0 | 0 | | 0 | 2 | 2 |
| MILD-762 | 72 | 0 | 1 | 0 | | 0 | | 0 | 0 | 0 | 0 | | 0 | 3 | 1 |
| MILD-764 | 41 | 1 | 0 | 1 | | 0 | | 0 | 0 | 0 | 0 | | 0 | 3 | 2 |
| MILD-765 | 61 | 1 | 1 | 0 | | 0 | | 1 | 0 | 0 | 0 | | 0 | 2 | 1 |
| MILD-767 | 61 | 0 | 0 | 0 | | 0 | | 0 | 0 | 0 | 0 | | 0 | 1 | 1 |
| MILD-769 | 36 | 0 | 0 | 0 | | 0 | | 0 | 0 | 0 | 0 | | 0 | 2 | 1 |
| MILD-774 | 63 | 0 | 1 | 0 | | 0 | | 0 | 1 | 0 | 0 | | 0 | 1 | 3 |
| MILD-776 | 33 | 1 | 0 | 0 | | 0 | | 0 | 0 | 0 | 0 | | 0 | 1 | 2 |
| MILD-777 | 53 | 1 | 0 | 0 | | 0 | | 0 | 0 | 1 | 0 | | 0 | 1 | 1 |
| MILD-779 | 47 | 0 | 1 | 0 | | 0 | | 0 | 0 | 0 | 0 | | 0 | 2 | 1 |
| MILD-783 | 68 | 1 | 1 | 0 | | 0 | | 0 | 0 | 0 | 0 | | 0 | 2 | 1 |
| MILD-784 | 39 | 0 | 0 | 0 | | 0 | | 0 | 0 | 0 | 0 | | 0 | 2 | 1 |
| MILD-789 | 75 | 1 | 1 | 1 | | 0 | | 1 | 0 | 1 | 0 | | 0 | 3 | 1 |
| MILD-791 | 54 | 0 | 0 | 0 | | 0 | | 0 | 0 | 0 | 0 | | 0 | 2 | 1 |
| MILD-795 | 56 | 0 | 1 | 1 | | 0 | | 0 | 0 | 0 | 0 | | 0 | 3 | 3 |
| MILD-797 | 67 | 0 | 0 | 1 | | 0 | | 0 | 0 | 0 | 0 | | 0 | 1 | 1 |
| MILD-798 | 58 | 0 | 1 | 0 | | 0 | | 0 | 1 | 0 | 0 | | 0 | 3 | 3 |
| MILD-799 | 68 | 1 | 0 | 0 | | 0 | | 0 | 0 | 0 | 0 | | 0 | 2 | 1 |
| MILD-800 | 69 | 1 | 0 | 0 | | 0 | | 0 | 0 | 0 | 0 | | 0 | 2 | 2 |
| MILD-803 | 50 | 0 | 1 | 0 | | 1 | | 0 | 0 | 0 | 0 | | 1 | 3 | 1 |
| MILD-804 | 50 | 1 | 0 | 0 | | 0 | | 0 | 0 | 0 | 0 | | 0 | 2 | 2 |
| MILD-810 | 75 | 1 | 1 | 0 | | 0 | | 0 | 0 | 0 | 0 | | 0 | 1 | 3 |
| MILD-815 | 82 | 0 | 1 | 0 | | 0 | | 0 | 0 | 1 | 0 | | 0 | 3 | 3 |
| MILD-828 | 18 | 0 | 0 | 0 | | 0 | | 0 | 0 | 0 | 0 | | 0 | 2 | 3 |
| MILD-830 | 76 | 1 | 1 | 1 | | 0 | | 0 | 0 | 0 | 1 | | 0 | 3 | 1 |
| MILD-832 | 43 | 0 | 0 | 0 | | 0 | | 0 | 0 | 0 | 0 | | 0 | 2 | 1 |
| MILD-836 | 61 | 0 | 0 | 0 | | 0 | | 0 | 0 | 0 | 0 | | 0 | 1 | 3 |
| MILD-843 | 69 | 0 | 1 | 0 | | 0 | | 0 | 0 | 0 | 0 | | 0 | 3 | 3 |
| MILD-845 | 63 | 0 | 1 | 0 | | 1 | | 0 | 0 | 1 | 0 | | 1 | 2 | 3 |
| MILD-850 | 70 | 1 | 1 | 0 | | 0 | | 0 | 0 | 0 | 0 | | 0 | 2 | 1 |
| MILD-851 | 51 | 1 | 0 | 0 | | 0 | | 0 | 0 | 1 | 0 | | 0 | 2 | 3 |
| MILD-859 | 19 | 1 | 0 | 0 | | 0 | | 0 | 0 | 0 | 0 | | 0 | 2 | 2 |
| MILD-868 | 62 | 1 | 1 | 1 | | 0 | | 0 | 0 | 0 | 0 | | 0 | 2 | 1 |
| MILD-876 | 14 | 1 | 0 | 0 | | 0 | | 0 | 0 | 0 | 0 | | 0 | 1 | 3 |
| MILD-877 | 34 | 1 | 1 | 0 | | 0 | | 0 | 0 | 0 | 0 | | 0 | 2 | 1 |
| MILD-887 | 50 | 0 | 1 | 0 | | 0 | | 0 | 0 | 0 | 0 | | 0 | 2 | 1 |
| MILD-890 | 61 | 0 | 1 | 0 | | 1 | | 0 | 0 | 0 | 0 | | 1 | 2 | 1 |
| MILD-897 | 80 | 0 | 0 | 0 | | 1 | | 0 | 0 | 0 | 0 | | 1 | 2 | 1 |
| MILD-898 | 61 | 1 | 1 | 1 | | 1 | | 1 | 1 | 0 | 0 | | 1 | 2 | 1 |
| MILD-899 | 40 | 0 | 0 | 0 | | 1 | | 0 | 0 | 0 | 0 | | 1 | 2 | 3 |
| MILD-901 | 63 | 1 | 1 | 1 | | 0 | | 0 | 0 | 0 | 0 | | 0 | 2 | 1 |
| MILD-902 | 23 | 1 | 0 | 0 | | 1 | | 0 | 0 | 0 | 0 | | 1 | 3 | 1 |
| MILD-903 | 60 | 0 | 1 | 1 | | 1 | | 0 | 1 | 0 | 0 | | 1 | 2 | 1 |
| MILD-917 | 83 | 1 | 1 | 0 | | 0 | | 0 | 0 | 0 | 0 | | 0 | 1 | 1 |
| MILD-918 | 79 | 1 | 1 | 1 | | 0 | | 0 | 0 | 0 | 0 | | 0 | 3 | 1 |
| MILD-920 | 32 | 0 | 0 | 0 | | 1 | | 0 | 0 | 0 | 0 | | 1 | 2 | 1 |
| MILD-921 | 64 | 1 | 1 | 1 | | 0 | | 0 | 0 | 0 | 0 | | 0 | 1 | 2 |
| MILD-924 | 70 | 1 | 1 | 0 | | 0 | | 0 | 1 | 1 | 0 | | 0 | 3 | 2 |
| MILD-926 | 76 | 1 | 0 | 0 | | 1 | | 0 | 0 | 0 | 0 | | 1 | 3 | 2 |
| MILD-928 | 57 | 1 | 0 | 0 | | 1 | | 0 | 0 | 0 | 0 | | 1 | 3 | 2 |
| MILD-929 | 68 | 0 | 0 | 0 | | 0 | | 0 | 0 | 0 | 0 | | 0 | 2 | 1 |
| MILD-932 | 68 | 0 | 0 | 0 | | 1 | | 0 | 0 | 0 | 0 | | 1 | 2 | 1 |

RSDs – Respiratory System Diseases. CVDs – Cardiovascular Diseases. NDDs – Neuropsychiatric Diseases or Disorders. ACKDs – Acute or Chronic Kidney Diseases. 1=No or Ausence; 0=Yes or Presence. ACE ID genotypes (II=1; ID=2; DD=3); ACE2 G8790A genotypes (GG=1, GA=2, AA=3 for female; G=1 and A=3 for male).

**SEVERE-COVID-19 GROUP**

| Patient Id | Age  (years) | Sex  (M=0  F=1) | Hypertension | Diabetes | RSDs | CVD | Obesity | NDDs | ACKDs | Smoking | ACE ID  (II=1; ID=2; DD=3) | ACE2 G8790A F: GG=1, GA=2, AA=3 M: G=1; A=3 |
| --- | --- | --- | --- | --- | --- | --- | --- | --- | --- | --- | --- | --- |
| SEVERE-105 | 67 | 1 | 0 | 0 | 0 | 0 | 0 | 0 | 0 | 0 | 3 | 1 |
| SEVERE-111 | 32 | 0 | 0 | 0 | 0 | 0 | 0 | 0 | 0 | 0 | 2 | 1 |
| SEVERE-113 | 60 | 1 | 1 | 1 | 0 | 0 | 0 | 0 | 0 | 0 | 3 | 1 |
| SEVERE-115 | 101 | 0 | 1 | 1 | 0 | 0 | 0 | 0 | 0 | 0 | 3 | 1 |
| SEVERE-116 | 68 | 0 | 0 | 0 | 0 | 0 | 0 | 0 | 0 | 0 | 3 | 1 |
| SEVERE-116 | 68 | 0 | 0 | 0 | 0 | 0 | 0 | 0 | 1 | 0 | 2 | 3 |
| SEVERE-118 | 72 | 1 | 1 | 0 | 1 | 0 | 0 | 0 | 0 | 1 | 2 | 2 |
| SEVERE-125 | 70 | 1 | 0 | 0 | 0 | 0 | 0 | 0 | 0 | 0 | 2 | 1 |
| SEVERE-125 | 70 | 1 | 0 | 0 | 0 | 0 | 0 | 0 | 0 | 0 | 1 | 1 |
| SEVERE-131 | 34 | 1 | 0 | 0 | 0 | 0 | 0 | 0 | 0 | 0 | 1 | 1 |
| SEVERE-135 | 63 | 1 | 0 | 0 | 0 | 0 | 0 | 0 | 1 | 0 | 2 | 1 |
| SEVERE-138 | 71 | 0 | 0 | 0 | 0 | 0 | 0 | 0 | 0 | 0 | 2 | 1 |
| SEVERE-140 | 57 | 1 | 0 | 0 | 0 | 0 | 0 | 0 | 0 | 0 | 1 | 1 |
| SEVERE-143 | 85 | 1 | 0 | 0 | 0 | 0 | 0 | 0 | 0 | 0 | 2 | 1 |
| SEVERE-144 | 27 | 1 | 0 | 0 | 0 | 0 | 0 | 0 | 0 | 0 | 1 | 1 |
| SEVERE-147 | 79 | 0 | 0 | 0 | 0 | 0 | 0 | 0 | 0 | 0 | 2 | 1 |
| SEVERE-151 | 41 | 0 | 0 | 0 | 0 | 0 | 0 | 0 | 0 | 0 | 3 | 1 |
| SEVERE-151 | 41 | 0 | 0 | 0 | 0 | 0 | 0 | 0 | 0 | 0 | 3 | 1 |
| SEVERE-152 | 40 | 0 | 0 | 0 | 0 | 0 | 0 | 0 | 0 | 0 | 2 | 1 |
| SEVERE-153 | 66 | 0 | 0 | 0 | 0 | 0 | 0 | 0 | 0 | 0 | 2 | 3 |
| SEVERE-155 | 64 | 0 | 0 | 0 | 0 | 0 | 0 | 0 | 0 | 0 | 1 | 1 |
| SEVERE-163 | 80 | 1 | 0 | 0 | 0 | 0 | 0 | 0 | 0 | 0 | 1 | 3 |
| SEVERE-167 | 58 | 0 | 0 | 0 | 0 | 0 | 0 | 0 | 0 | 0 | 1 | 1 |
| SEVERE-169 | 68 | 0 | 0 | 0 | 0 | 1 | 0 | 0 | 0 | 0 | 2 | 1 |
| SEVERE-171 | 59 | 0 | 0 | 0 | 0 | 0 | 0 | 0 | 1 | 0 | 1 | 3 |
| SEVERE-187 | 68 | 0 | 0 | 0 | 0 | 0 | 0 | 0 | 0 | 0 | 2 | 1 |
| SEVERE-191 | 81 | 1 | 0 | 0 | 0 | 0 | 0 | 0 | 0 | 0 | 3 | 1 |
| SEVERE-194 | 95 | 0 | 0 | 0 | 0 | 0 | 0 | 0 | 0 | 0 | 2 | 1 |
| SEVERE-200 | 56 | 0 | 1 | 0 | 0 | 0 | 0 | 0 | 0 | 0 | 1 | 1 |
| SEVERE-200 | 56 | 0 | 1 | 0 | 0 | 0 | 0 | 0 | 0 | 0 | 3 | 1 |
| SEVERE-204 | 78 | 1 | 0 | 0 | 0 | 0 | 0 | 0 | 0 | 0 | 1 | 2 |
| SEVERE-210 | 61 | 0 | 1 | 1 | 0 | 0 | 1 | 0 | 0 | 0 | 1 | 3 |
| SEVERE-224 | 46 | 0 | 0 | 0 | 1 | 0 | 1 | 0 | 0 | 0 | 2 | 1 |
| SEVERE-225 | 79 | 0 | 0 | 0 | 0 | 0 | 0 | 0 | 0 | 0 | 1 | 1 |
| SEVERE-232 | 70 | 0 | 0 | 0 | 0 | 0 | 0 | 0 | 0 | 0 | 2 | 1 |
| SEVERE-233 | 67 | 1 | 0 | 0 | 0 | 0 | 0 | 0 | 0 | 0 | 2 | 1 |
| SEVERE-234 | 58 | 1 | 0 | 0 | 0 | 0 | 0 | 0 | 0 | 0 | 3 | 3 |
| SEVERE-237 | 84 | 1 | 0 | 0 | 0 | 0 | 0 | 0 | 0 | 0 | 2 | 2 |
| SEVERE-239 | 60 | 1 | 1 | 1 | 0 | 1 | 0 | 0 | 0 | 0 | 3 | 1 |
| SEVERE-243 | 60 | 1 | 0 | 0 | 0 | 0 | 0 | 0 | 0 | 0 | 2 | 3 |
| SEVERE-248 | 60 | 0 | 0 | 0 | 0 | 0 | 0 | 0 | 0 | 0 | 3 | 3 |
| SEVERE-253 | 73 | 0 | 1 | 1 | 0 | 0 | 0 | 0 | 0 | 0 | 2 | 1 |
| SEVERE-256 | 72 | 0 | 0 | 0 | 0 | 0 | 0 | 0 | 0 | 0 | 2 | 3 |
| SEVERE-259 | 75 | 0 | 1 | 0 | 1 | 0 | 0 | 0 | 0 | 1 | 2 | 3 |
| SEVERE-260 | 51 | 1 | 0 | 0 | 0 | 0 | 0 | 0 | 0 | 0 | 2 | 2 |
| SEVERE-265 | 57 | 1 | 1 | 1 | 0 | 0 | 0 | 0 | 0 | 0 | 1 | 1 |
| SEVERE-282 | 52 | 1 | 0 | 0 | 1 | 0 | 1 | 0 | 0 | 1 | 2 | 1 |
| SEVERE-300 | 63 | 0 | 1 | 1 | 0 | 0 | 0 | 0 | 0 | 0 | 1 | 1 |
| SEVERE-303 | 89 | 0 | 1 | 0 | 0 | 0 | 0 | 0 | 0 | 0 | 2 | 1 |
| SEVERE-304 | 61 | 1 | 1 | 0 | 0 | 0 | 0 | 0 | 1 | 0 | 2 | 2 |
| SEVERE-306 | 89 | 0 | 0 | 0 | 0 | 1 | 0 | 1 | 0 | 0 | 2 | 1 |
| SEVERE-307 | 62 | 0 | 0 | 1 | 0 | 0 | 0 | 0 | 0 | 0 | 2 | 1 |
| SEVERE-309 | 83 | 1 | 1 | 0 | 0 | 0 | 1 | 0 | 0 | 0 | 1 | 1 |
| SEVERE-310 | 82 | 0 | 0 | 0 | 0 | 0 | 0 | 0 | 0 | 0 | 2 | 1 |
| SEVERE-313 | 71 | 0 | 0 | 0 | 0 | 0 | 0 | 0 | 0 | 0 | 1 | 1 |
| SEVERE-315 | 86 | 0 | 0 | 0 | 1 | 0 | 0 | 0 | 0 | 0 | 2 | 1 |
| SEVERE-318 | 64 | 1 | 0 | 0 | 0 | 0 | 0 | 1 | 0 | 0 | 3 | 2 |
| SEVERE-319 | 59 | 0 | 0 | 0 | 0 | 0 | 0 | 1 | 0 | 0 | 3 | 3 |
| SEVERE-323 | 53 | 0 | 0 | 0 | 0 | 0 | 0 | 0 | 0 | 0 | 2 | 1 |
| SEVERE-327 | 81 | 0 | 0 | 0 | 1 | 1 | 0 | 0 | 0 | 0 | 3 | 1 |
| SEVERE-329 | 71 | 0 | 0 | 0 | 0 | 0 | 0 | 0 | 0 | 0 | 2 | 1 |
| SEVERE-333 | 93 | 0 | 0 | 0 | 0 | 0 | 0 | 0 | 0 | 0 | 2 | 1 |
| SEVERE-343 | 63 | 0 | 0 | 0 | 0 | 0 | 0 | 0 | 0 | 0 | 2 | 1 |
| SEVERE-356 | 84 | 0 | 0 | 0 | 0 | 0 | 0 | 0 | 0 | 0 | 2 | 1 |
| SEVERE-357 | 72 | 1 | 0 | 0 | 0 | 0 | 0 | 0 | 0 | 0 | 2 | 1 |
| SEVERE-360 | 57 | 0 | 0 | 0 | 0 | 0 | 0 | 0 | 0 | 0 | 1 | 1 |
| SEVERE-366 | 70 | 0 | 0 | 0 | 0 | 0 | 0 | 0 | 0 | 0 | 3 | 1 |
| SEVERE-375 | 66 | 0 | 0 | 0 | 0 | 0 | 0 | 0 | 0 | 0 | 1 | 3 |
| SEVERE-376 | 61 | 0 | 0 | 0 | 0 | 0 | 0 | 0 | 1 | 0 | 1 | 1 |
| SEVERE-381 | 61 | 0 | 1 | 0 | 0 | 0 | 1 | 0 | 0 | 0 | 1 | 1 |
| SEVERE-383 | 53 | 0 | 0 | 0 | 0 | 0 | 0 | 0 | 0 | 0 | 3 | 1 |
| SEVERE-384 | 53 | 0 | 1 | 1 | 0 | 0 | 0 | 1 | 0 | 0 | 2 | 1 |
| SEVERE-390 | 64 | 1 | 0 | 0 | 0 | 0 | 0 | 0 | 0 | 0 | 3 | 2 |
| SEVERE-392 | 70 | 1 | 1 | 1 | 0 | 0 | 0 | 0 | 0 | 0 | 2 | 1 |
| SEVERE-394 | 67 | 0 | 0 | 0 | 0 | 0 | 0 | 0 | 0 | 0 | 2 | 1 |
| SEVERE-398 | 86 | 0 | 0 | 0 | 0 | 0 | 0 | 0 | 0 | 0 | 2 | 1 |
| SEVERE-399 | 68 | 0 | 0 | 0 | 0 | 0 | 0 | 0 | 0 | 0 | 2 | 1 |
| SEVERE-400 | 79 | 0 | 1 | 1 | 0 | 0 | 0 | 1 | 0 | 0 | 2 | 1 |
| SEVERE-401 | 60 | 0 | 0 | 0 | 0 | 0 | 0 | 0 | 1 | 0 | 1 | 3 |
| SEVERE-402 | 66 | 0 | 1 | 1 | 0 | 0 | 0 | 0 | 0 | 0 | 2 | 1 |
| SEVERE-406 | 80 | 0 | 0 | 0 | 0 | 0 | 0 | 1 | 0 | 0 | 2 | 1 |
| SEVERE-411 | 74 | 1 | 1 | 1 | 0 | 0 | 0 | 0 | 0 | 0 | 3 | 1 |
| SEVERE-414 | 71 | 1 | 0 | 0 | 0 | 0 | 0 | 0 | 0 | 0 | 1 | 1 |
| SEVERE-421 | 60 | 1 | 0 | 0 | 0 | 1 | 0 | 0 | 0 | 0 | 2 | 1 |
| SEVERE-424 | 71 | 1 | 0 | 0 | 0 | 0 | 0 | 0 | 0 | 0 | 2 | 2 |
| SEVERE-432 | 68 | 1 | 0 | 0 | 0 | 0 | 0 | 0 | 0 | 0 | 2 | 2 |
| SEVERE-434 | 78 | 1 | 1 | 0 | 1 | 0 | 0 | 0 | 0 | 0 | 2 | 1 |
| SEVERE-439 | 64 | 0 | 0 | 0 | 0 | 0 | 0 | 0 | 0 | 0 | 1 | 1 |
| SEVERE-451 | 67 | 1 | 1 | 1 | 0 | 0 | 1 | 0 | 0 | 0 | 1 | 1 |
| SEVERE-452 | 53 | 0 | 0 | 0 | 0 | 0 | 0 | 0 | 0 | 0 | 3 | 1 |
| SEVERE-453 | 84 | 0 | 0 | 0 | 0 | 0 | 0 | 0 | 0 | 0 | 1 | 1 |
| SEVERE-459 | 77 | 0 | 0 | 0 | 0 | 1 | 0 | 0 | 0 | 0 | 2 | 1 |
| SEVERE-461 | 63 | 0 | 0 | 0 | 0 | 0 | 0 | 0 | 0 | 0 | 2 | 1 |
| SEVERE-462 | 65 | 0 | 1 | 1 | 1 | 0 | 0 | 0 | 0 | 0 | 2 | 3 |
| SEVERE-467 | 72 | 0 | 0 | 0 | 0 | 0 | 0 | 0 | 0 | 0 | 2 | 1 |
| SEVERE-469 | 60 | 0 | 0 | 0 | 0 | 0 | 0 | 0 | 0 | 0 | 3 | 3 |
| SEVERE-472 | 72 | 0 | 0 | 0 | 0 | 0 | 0 | 0 | 0 | 0 | 2 | 1 |
| SEVERE-474 | 78 | 0 | 0 | 0 | 0 | 0 | 0 | 0 | 0 | 0 | 3 | 1 |
| SEVERE-480 | 83 | 0 | 0 | 0 | 0 | 0 | 0 | 0 | 0 | 0 | 3 | 1 |
| SEVERE-481 | 84 | 1 | 1 | 1 | 0 | 0 | 0 | 1 | 0 | 0 | 1 | 1 |
| SEVERE-483 | 59 | 0 | 1 | 0 | 0 | 0 | 0 | 0 | 0 | 0 | 2 | 1 |
| SEVERE-485 | 65 | 1 | 1 | 1 | 1 | 0 | 0 | 0 | 0 | 0 | 1 | 1 |
| SEVERE-486 | 57 | 1 | 0 | 0 | 0 | 0 | 0 | 0 | 0 | 0 | 2 | 1 |
| SEVERE-488 | 53 | 1 | 0 | 0 | 0 | 0 | 0 | 0 | 1 | 0 | 3 | 1 |
| SEVERE-491 | 71 | 1 | 1 | 1 | 0 | 1 | 0 | 0 | 0 | 0 | 2 | 1 |
| SEVERE-494 | 63 | 0 | 1 | 1 | 0 | 0 | 0 | 0 | 1 | 0 | 1 | 1 |
| SEVERE-497 | 84 | 1 | 0 | 0 | 1 | 0 | 0 | 0 | 0 | 1 | 2 | 1 |
| SEVERE-499 | 70 | 0 | 0 | 0 | 1 | 0 | 0 | 0 | 0 | 0 | 3 | 3 |
| SEVERE-502 | 67 | 1 | 0 | 0 | 1 | 0 | 0 | 0 | 0 | 0 | 2 | 1 |
| SEVERE-505 | 55 | 1 | 0 | 0 | 0 | 0 | 0 | 0 | 0 | 0 | 3 | 1 |
| SEVERE-506 | 74 | 1 | 0 | 0 | 0 | 0 | 0 | 0 | 0 | 0 | 2 | 3 |
| SEVERE-512 | 57 | 0 | 0 | 1 | 0 | 0 | 0 | 0 | 0 | 0 | 3 | 1 |
| SEVERE-513 | 61 | 0 | 1 | 0 | 1 | 0 | 0 | 0 | 0 | 1 | 3 | 1 |
| SEVERE-515 | 43 | 1 | 0 | 1 | 0 | 0 | 1 | 0 | 0 | 0 | 1 | 1 |
| SEVERE-519 | 73 | 1 | 0 | 0 | 0 | 0 | 0 | 0 | 0 | 0 | 1 | 1 |
| SEVERE-521 | 77 | 1 | 1 | 0 | 1 | 0 | 0 | 0 | 0 | 0 | 2 | 2 |
| SEVERE-538 | 62 | 1 | 0 | 0 | 0 | 0 | 1 | 1 | 0 | 0 | 2 | 1 |
| SEVERE-542 | 67 | 0 | 0 | 1 | 0 | 0 | 0 | 1 | 0 | 0 | 1 | 3 |
| SEVERE-544 | 84 | 1 | 1 | 1 | 0 | 1 | 0 | 0 | 0 | 0 | 2 | 1 |
| SEVERE-547 | 86 | 0 | 0 | 0 | 1 | 0 | 0 | 0 | 0 | 0 | 1 | 1 |
| SEVERE-548 | 70 | 1 | 1 | 1 | 0 | 0 | 0 | 0 | 0 | 0 | 3 | 2 |
| SEVERE-550 | 82 | 1 | 1 | 0 | 1 | 1 | 0 | 1 | 0 | 1 | 3 | 1 |
| SEVERE-553 | 77 | 0 | 1 | 1 | 0 | 0 | 0 | 0 | 0 | 0 | 3 | 1 |
| SEVERE-554 | 85 | 1 | 0 | 0 | 0 | 0 | 0 | 1 | 0 | 0 | 2 | 1 |
| SEVERE-557 | 46 | 1 | 1 | 0 | 0 | 1 | 1 | 0 | 0 | 0 | 3 | 1 |
| SEVERE-559 | 41 | 0 | 0 | 0 | 0 | 0 | 0 | 0 | 0 | 0 | 2 | 1 |
| SEVERE-562 | 97 | 1 | 1 | 1 | 0 | 0 | 0 | 1 | 0 | 0 | 2 | 2 |
| SEVERE-563 | 56 | 1 | 0 | 0 | 0 | 0 | 1 | 0 | 0 | 0 | 3 | 1 |
| SEVERE-565 | 58 | 1 | 1 | 1 | 0 | 0 | 0 | 0 | 0 | 0 | 2 | 1 |
| SEVERE-567 | 62 | 0 | 1 | 1 | 0 | 0 | 0 | 0 | 0 | 0 | 2 | 1 |
| SEVERE-568 | 78 | 1 | 1 | 0 | 0 | 0 | 0 | 0 | 0 | 0 | 2 | 2 |
| SEVERE-570 | 71 | 1 | 0 | 0 | 0 | 0 | 0 | 1 | 0 | 0 | 3 | 1 |
| SEVERE-573 | 48 | 0 | 0 | 0 | 1 | 0 | 0 | 0 | 0 | 0 | 2 | 1 |
| SEVERE-574 | 82 | 0 | 1 | 0 | 1 | 1 | 0 | 0 | 0 | 1 | 2 | 3 |
| SEVERE-581 | 38 | 0 | 1 | 0 | 0 | 0 | 0 | 0 | 0 | 0 | 2 | 3 |
| SEVERE-584 | 85 | 0 | 1 | 1 | 1 | 0 | 0 | 0 | 0 | 0 | 3 | 1 |
| SEVERE-587 | 46 | 0 | 0 | 0 | 0 | 0 | 0 | 0 | 0 | 0 | 1 | 3 |
| SEVERE-588 | 67 | 0 | 0 | 0 | 1 | 0 | 0 | 0 | 0 | 0 | 2 | 1 |
| SEVERE-591 | 57 | 1 | 0 | 1 | 0 | 0 | 0 | 0 | 0 | 0 | 1 | 1 |
| SEVERE-592 | 54 | 1 | 0 | 0 | 1 | 0 | 1 | 0 | 0 | 0 | 2 | 1 |
| SEVERE-593 | 67 | 1 | 0 | 0 | 0 | 0 | 0 | 0 | 0 | 0 | 2 | 2 |
| SEVERE-594 | 69 | 1 | 0 | 0 | 0 | 0 | 0 | 1 | 0 | 0 | 2 | 1 |
| SEVERE-595 | 59 | 0 | 1 | 0 | 0 | 0 | 0 | 0 | 0 | 0 | 3 | 3 |
| SEVERE-596 | 53 | 0 | 1 | 0 | 0 | 0 | 0 | 0 | 0 | 0 | 2 | 1 |
| SEVERE-599 | 70 | 1 | 1 | 0 | 1 | 1 | 0 | 0 | 0 | 0 | 2 | 1 |
| SEVERE-601 | 79 | 0 | 0 | 0 | 0 | 0 | 0 | 0 | 0 | 0 | 1 | 1 |
| SEVERE-602 | 73 | 0 | 1 | 1 | 0 | 0 | 1 | 0 | 0 | 0 | 3 | 3 |
| SEVERE-603 | 42 | 1 | 0 | 0 | 0 | 0 | 0 | 0 | 0 | 0 | 2 | 1 |
| SEVERE-604 | 34 | 1 | 0 | 1 | 0 | 0 | 0 | 0 | 0 | 0 | 2 | 2 |
| SEVERE-606 | 45 | 1 | 0 | 0 | 0 | 0 | 0 | 0 | 0 | 0 | 3 | 1 |
| SEVERE-607 | 67 | 0 | 1 | 0 | 0 | 0 | 0 | 0 | 0 | 0 | 2 | 1 |
| SEVERE-608 | 84 | 0 | 0 | 0 | 0 | 0 | 0 | 1 | 0 | 0 | 3 | 1 |
| SEVERE-610 | 40 | 1 | 0 | 1 | 0 | 0 | 0 | 1 | 0 | 0 | 2 | 2 |
| SEVERE-611 | 40 | 0 | 0 | 0 | 0 | 0 | 0 | 0 | 0 | 0 | 2 | 1 |
| SEVERE-612 | 89 | 1 | 0 | 1 | 1 | 0 | 0 | 0 | 0 | 1 | 2 | 2 |
| SEVERE-613 | 87 | 0 | 1 | 0 | 0 | 0 | 0 | 0 | 0 | 0 | 3 | 1 |
| SEVERE-616 | 54 | 0 | 1 | 0 | 0 | 1 | 0 | 1 | 0 | 0 | 2 | 1 |
| SEVERE-622 | 43 | 1 | 1 | 0 | 0 | 0 | 1 | 0 | 0 | 0 | 2 | 2 |
| SEVERE-625 | 69 | 1 | 1 | 1 | 0 | 0 | 0 | 0 | 0 | 0 | 2 | 2 |
| SEVERE-630 | 76 | 1 | 1 | 1 | 0 | 0 | 0 | 0 | 0 | 0 | 2 | 3 |
| SEVERE-632 | 70 | 0 | 1 | 1 | 1 | 0 | 0 | 0 | 1 | 1 | 1 | 1 |
| SEVERE-636 | 48 | 0 | 0 | 0 | 0 | 0 | 0 | 0 | 0 | 0 | 1 | 1 |
| SEVERE-637 | 71 | 0 | 1 | 0 | 0 | 0 | 0 | 0 | 0 | 0 | 2 | 1 |
| SEVERE-641 | 67 | 0 | 1 | 0 | 0 | 0 | 0 | 0 | 0 | 0 | 2 | 1 |
| SEVERE-642 | 89 | 1 | 1 | 1 | 0 | 0 | 0 | 0 | 0 | 0 | 3 | 1 |
| SEVERE-643 | 87 | 0 | 0 | 0 | 1 | 0 | 0 | 0 | 0 | 1 | 2 | 3 |
| SEVERE-645 | 40 | 0 | 0 | 0 | 0 | 0 | 0 | 0 | 0 | 0 | 3 | 1 |
| SEVERE-646 | 77 | 0 | 0 | 0 | 0 | 0 | 0 | 0 | 0 | 0 | 1 | 1 |
| SEVERE-647 | 72 | 1 | 1 | 1 | 1 | 1 | 0 | 0 | 0 | 1 | 3 | 3 |
| SEVERE-649 | 39 | 1 | 0 | 0 | 0 | 0 | 0 | 0 | 0 | 0 | 3 | 1 |
| SEVERE-654 | 51 | 1 | 0 | 0 | 0 | 0 | 0 | 1 | 0 | 0 | 2 | 1 |
| SEVERE-655 | 60 | 0 | 1 | 0 | 1 | 0 | 0 | 0 | 0 | 1 | 2 | 1 |
| SEVERE-656 | 43 | 0 | 0 | 1 | 1 | 0 | 0 | 0 | 0 | 1 | 2 | 3 |
| SEVERE-657 | 71 | 0 | 1 | 0 | 0 | 1 | 0 | 0 | 0 | 0 | 2 | 1 |
| SEVERE-659 | 66 | 1 | 1 | 0 | 0 | 0 | 0 | 0 | 0 | 0 | 2 | 1 |
| SEVERE-659 | 66 | 1 | 1 | 0 | 0 | 0 | 0 | 0 | 0 | 0 | 2 | 2 |
| SEVERE-662 | 90 | 1 | 1 | 0 | 1 | 0 | 0 | 1 | 0 | 1 | 1 | 1 |
| SEVERE-665 | 73 | 0 | 0 | 0 | 0 | 0 | 0 | 0 | 0 | 0 | 2 | 1 |
| SEVERE-666 | 65 | 1 | 1 | 1 | 0 | 0 | 1 | 0 | 0 | 0 | 2 | 2 |
| SEVERE-669 | 82 | 1 | 1 | 1 | 0 | 0 | 0 | 0 | 0 | 0 | 3 | 1 |
| SEVERE-672 | 70 | 0 | 0 | 0 | 1 | 0 | 0 | 0 | 0 | 1 | 3 | 3 |
| SEVERE-673 | 44 | 1 | 0 | 0 | 0 | 0 | 0 | 1 | 0 | 0 | 1 | 1 |
| SEVERE-674 | 52 | 0 | 0 | 0 | 0 | 0 | 0 | 0 | 0 | 0 | 3 | 1 |
| SEVERE-676 | 62 | 0 | 1 | 0 | 0 | 0 | 0 | 1 | 0 | 0 | 2 | 1 |
| SEVERE-680 | 78 | 1 | 1 | 0 | 0 | 0 | 0 | 0 | 0 | 0 | 3 | 2 |
| SEVERE-681 | 64 | 0 | 1 | 0 | 1 | 0 | 0 | 0 | 0 | 0 | 1 | 3 |
| SEVERE-681 | 64 | 0 | 1 | 0 | 1 | 0 | 0 | 0 | 0 | 0 | 1 | 3 |
| SEVERE-683 | 67 | 1 | 1 | 1 | 1 | 1 | 0 | 0 | 0 | 0 | 2 | 2 |
| SEVERE-685 | 67 | 0 | 1 | 1 | 0 | 0 | 0 | 0 | 0 | 0 | 2 | 1 |
| SEVERE-686 | 45 | 0 | 1 | 1 | 0 | 0 | 1 | 0 | 0 | 0 | 1 | 1 |
| SEVERE-689 | 50 | 0 | 0 | 0 | 0 | 0 | 0 | 0 | 0 | 0 | 2 | 3 |
| SEVERE-690 | 69 | 0 | 0 | 0 | 0 | 0 | 0 | 0 | 0 | 0 | 3 | 1 |
| SEVERE-691 | 73 | 0 | 1 | 1 | 0 | 0 | 0 | 1 | 0 | 0 | 1 | 3 |
| SEVERE-693 | 85 | 1 | 1 | 0 | 1 | 0 | 0 | 0 | 0 | 0 | 2 | 2 |
| SEVERE-696 | 83 | 0 | 1 | 1 | 0 | 0 | 0 | 0 | 0 | 0 | 2 | 1 |
| SEVERE-697 | 78 | 0 | 1 | 0 | 0 | 0 | 0 | 1 | 0 | 0 | 2 | 1 |
| SEVERE-698 | 79 | 0 | 0 | 0 | 0 | 0 | 0 | 0 | 0 | 0 | 2 | 1 |
| SEVERE-702 | 48 | 0 | 1 | 1 | 0 | 0 | 0 | 0 | 0 | 0 | 3 | 1 |
| SEVERE-703 | 40 | 1 | 0 | 0 | 0 | 0 | 0 | 0 | 0 | 0 | 2 | 3 |
| SEVERE-704 | 58 | 1 | 1 | 0 | 0 | 0 | 0 | 0 | 0 | 0 | 3 | 2 |
| SEVERE-711 | 50 | 0 | 0 | 1 | 0 | 0 | 0 | 0 | 0 | 0 | 3 | 1 |
| SEVERE-714 | 77 | 1 | 1 | 1 | 0 | 0 | 0 | 0 | 0 | 0 | 2 | 3 |
| SEVERE-715 | 64 | 0 | 1 | 1 | 0 | 0 | 0 | 0 | 0 | 0 | 3 | 1 |
| SEVERE-718 | 61 | 1 | 1 | 0 | 0 | 0 | 0 | 0 | 0 | 0 | 2 | 2 |
| SEVERE-720 | 96 | 0 | 0 | 0 | 1 | 0 | 0 | 0 | 0 | 0 | 3 | 1 |
| SEVERE-726 | 87 | 0 | 1 | 0 | 1 | 1 | 0 | 0 | 0 | 0 | 2 | 1 |
| SEVERE-729 | 82 | 1 | 0 | 0 | 0 | 0 | 0 | 0 | 1 | 0 | 3 | 2 |
| SEVERE-731 | 98 | 0 | 1 | 0 | 0 | 0 | 0 | 0 | 0 | 0 | 2 | 1 |
| SEVERE-737 | 50 | 1 | 0 | 0 | 0 | 0 | 0 | 0 | 0 | 0 | 2 | 1 |
| SEVERE-743 | 21 | 0 | 0 | 0 | 0 | 0 | 0 | 0 | 0 | 0 | 2 | 3 |
| SEVERE-746 | 70 | 1 | 1 | 0 | 1 | 0 | 0 | 0 | 0 | 0 | 3 | 1 |
| SEVERE-748 | 77 | 1 | 0 | 0 | 0 | 0 | 0 | 0 | 0 | 0 | 1 | 1 |
| SEVERE-756 | 65 | 0 | 1 | 0 | 0 | 0 | 0 | 0 | 0 | 0 | 3 | 3 |
| SEVERE-763 | 73 | 0 | 1 | 0 | 1 | 0 | 0 | 1 | 0 | 1 | 1 | 1 |
| SEVERE-766 | 84 | 0 | 1 | 0 | 1 | 1 | 0 | 0 | 1 | 0 | 2 | 3 |
| SEVERE-768 | 59 | 0 | 0 | 0 | 0 | 0 | 0 | 0 | 0 | 0 | 2 | 1 |
| SEVERE-770 | 52 | 0 | 1 | 0 | 0 | 0 | 0 | 0 | 0 | 0 | 2 | 1 |
| SEVERE-772 | 38 | 0 | 0 | 0 | 0 | 0 | 0 | 0 | 0 | 0 | 2 | 3 |
| SEVERE-775 | 67 | 0 | 1 | 1 | 0 | 0 | 0 | 0 | 0 | 0 | 1 | 1 |
| SEVERE-778 | 66 | 0 | 1 | 0 | 0 | 0 | 0 | 0 | 0 | 0 | 2 | 1 |
| SEVERE-781 | 79 | 0 | 1 | 1 | 0 | 0 | 0 | 0 | 0 | 0 | 3 | 1 |
| SEVERE-782 | 66 | 0 | 0 | 0 | 0 | 0 | 0 | 0 | 0 | 0 | 1 | 3 |
| SEVERE-785 | 82 | 1 | 0 | 0 | 1 | 0 | 0 | 1 | 0 | 0 | 2 | 2 |
| SEVERE-786 | 91 | 0 | 1 | 0 | 0 | 0 | 0 | 0 | 0 | 0 | 2 | 1 |
| SEVERE-790 | 98 | 0 | 1 | 0 | 0 | 0 | 0 | 0 | 0 | 0 | 2 | 1 |
| SEVERE-793 | 80 | 1 | 1 | 1 | 0 | 0 | 0 | 0 | 0 | 0 | 3 | 2 |
| SEVERE-801 | 46 | 1 | 0 | 0 | 1 | 0 | 0 | 0 | 0 | 0 | 2 | 1 |
| SEVERE-802 | 71 | 1 | 1 | 0 | 0 | 0 | 0 | 0 | 0 | 0 | 1 | 2 |
| SEVERE-805 | 60 | 1 | 1 | 0 | 0 | 1 | 0 | 0 | 0 | 0 | 2 | 1 |
| SEVERE-806 | 54 | 0 | 0 | 1 | 1 | 0 | 0 | 1 | 0 | 1 | 3 | 1 |
| SEVERE-808 | 70 | 1 | 1 | 1 | 0 | 0 | 0 | 0 | 0 | 0 | 3 | 2 |
| SEVERE-811 | 40 | 0 | 0 | 0 | 0 | 0 | 0 | 1 | 0 | 0 | 3 | 3 |
| SEVERE-812 | 49 | 1 | 0 | 0 | 0 | 0 | 0 | 0 | 0 | 0 | 3 | 1 |
| SEVERE-814 | 55 | 0 | 0 | 0 | 0 | 0 | 0 | 0 | 0 | 0 | 2 | 1 |
| SEVERE-816 | 46 | 0 | 1 | 1 | 0 | 0 | 1 | 0 | 0 | 0 | 3 | 1 |
| SEVERE-818 | 87 | 0 | 0 | 0 | 0 | 0 | 0 | 1 | 0 | 0 | 2 | 1 |
| SEVERE-823 | 55 | 0 | 1 | 0 | 0 | 0 | 0 | 0 | 0 | 0 | 3 | 1 |
| SEVERE-824 | 66 | 0 | 0 | 0 | 0 | 0 | 0 | 0 | 0 | 0 | 1 | 1 |
| SEVERE-825 | 71 | 0 | 1 | 0 | 1 | 0 | 0 | 0 | 0 | 1 | 3 | 1 |
| SEVERE-826 | 81 | 0 | 1 | 0 | 1 | 0 | 0 | 0 | 0 | 1 | 2 | 1 |
| SEVERE-827 | 32 | 0 | 0 | 0 | 0 | 0 | 0 | 0 | 0 | 0 | 2 | 1 |
| SEVERE-829 | 67 | 1 | 0 | 0 | 0 | 0 | 1 | 0 | 0 | 0 | 2 | 2 |
| SEVERE-831 | 60 | 0 | 1 | 1 | 0 | 0 | 0 | 0 | 0 | 0 | 2 | 1 |
| SEVERE-834 | 62 | 1 | 1 | 1 | 0 | 0 | 0 | 1 | 0 | 0 | 3 | 2 |
| SEVERE-837 | 55 | 1 | 0 | 0 | 0 | 0 | 0 | 0 | 0 | 0 | 3 | 3 |
| SEVERE-838 | 80 | 0 | 1 | 1 | 1 | 0 | 0 | 0 | 1 | 0 | 2 | 1 |
| SEVERE-839 | 70 | 0 | 0 | 0 | 0 | 0 | 0 | 1 | 0 | 0 | 2 | 1 |
| SEVERE-841 | 65 | 1 | 1 | 1 | 1 | 1 | 0 | 1 | 0 | 1 | 3 | 1 |
| SEVERE-842 | 50 | 0 | 0 | 0 | 0 | 0 | 0 | 0 | 0 | 0 | 3 | 1 |
| SEVERE-846 | 71 | 0 | 1 | 0 | 1 | 1 | 0 | 0 | 0 | 1 | 3 | 3 |
| SEVERE-847 | 60 | 1 | 1 | 0 | 0 | 0 | 0 | 0 | 0 | 0 | 2 | 1 |
| SEVERE-849 | 48 | 1 | 1 | 0 | 0 | 0 | 0 | 0 | 1 | 0 | 3 | 1 |
| SEVERE-852 | 71 | 1 | 1 | 0 | 0 | 0 | 1 | 0 | 0 | 0 | 1 | 1 |
| SEVERE-855 | 50 | 0 | 1 | 0 | 0 | 0 | 1 | 0 | 0 | 0 | 2 | 1 |
| SEVERE-856 | 69 | 0 | 1 | 0 | 0 | 0 | 0 | 1 | 0 | 0 | 2 | 1 |
| SEVERE-857 | 83 | 0 | 1 | 0 | 0 | 1 | 0 | 0 | 0 | 0 | 3 | 1 |
| SEVERE-858 | 74 | 1 | 1 | 1 | 0 | 1 | 0 | 0 | 0 | 0 | 2 | 2 |
| SEVERE-860 | 88 | 1 | 1 | 1 | 0 | 0 | 0 | 1 | 0 | 0 | 2 | 1 |
| SEVERE-861 | 86 | 1 | 0 | 0 | 0 | 0 | 0 | 0 | 1 | 0 | 3 | 1 |
| SEVERE-862 | 24 | 0 | 0 | 0 | 0 | 0 | 0 | 0 | 0 | 0 | 2 | 1 |
| SEVERE-863 | 77 | 0 | 0 | 0 | 0 | 1 | 0 | 0 | 0 | 0 | 2 | 3 |
| SEVERE-866 | 66 | 0 | 0 | 1 | 1 | 0 | 0 | 0 | 0 | 1 | 1 | 1 |
| SEVERE-867 | 44 | 0 | 0 | 0 | 0 | 0 | 0 | 0 | 0 | 0 | 2 | 1 |
| SEVERE-871 | 42 | 0 | 1 | 0 | 0 | 0 | 0 | 0 | 1 | 0 | 3 | 1 |
| SEVERE-872 | 70 | 0 | 0 | 0 | 1 | 1 | 0 | 0 | 0 | 0 | 2 | 1 |
| SEVERE-874 | 67 | 0 | 1 | 0 | 0 | 0 | 0 | 0 | 0 | 0 | 2 | 1 |
| SEVERE-875 | 52 | 0 | 0 | 1 | 0 | 0 | 0 | 0 | 0 | 0 | 1 | 1 |
| SEVERE-881 | 52 | 0 | 0 | 0 | 1 | 0 | 0 | 0 | 0 | 1 | 2 | 1 |
| SEVERE-884 | 74 | 0 | 0 | 1 | 1 | 0 | 0 | 0 | 0 | 1 | 2 | 3 |
| SEVERE-885 | 19 | 1 | 0 | 0 | 0 | 0 | 0 | 1 | 0 | 0 | 2 | 2 |
| SEVERE-886 | 72 | 1 | 1 | 1 | 0 | 0 | 0 | 0 | 0 | 0 | 2 | 1 |
| SEVERE-888 | 77 | 0 | 0 | 0 | 0 | 1 | 0 | 0 | 0 | 0 | 2 | 3 |
| SEVERE-889 | 84 | 1 | 0 | 0 | 0 | 0 | 0 | 1 | 0 | 0 | 2 | 1 |
| SEVERE-891 | 64 | 0 | 0 | 0 | 1 | 0 | 0 | 0 | 0 | 1 | 3 | 1 |
| SEVERE-892 | 67 | 1 | 1 | 0 | 0 | 1 | 1 | 0 | 0 | 0 | 2 | 2 |
| SEVERE-893 | 39 | 0 | 0 | 0 | 0 | 0 | 0 | 1 | 0 | 0 | 2 | 3 |
| SEVERE-895 | 63 | 1 | 0 | 1 | 0 | 1 | 0 | 0 | 0 | 0 | 1 | 1 |
| SEVERE-896 | 21 | 0 | 0 | 0 | 0 | 0 | 0 | 0 | 0 | 0 | 2 | 3 |
| SEVERE-904 | 58 | 1 | 0 | 1 | 0 | 1 | 0 | 1 | 0 | 0 | 3 | 1 |
| SEVERE-905 | 71 | 0 | 0 | 0 | 0 | 0 | 0 | 0 | 0 | 0 | 2 | 1 |
| SEVERE-906 | 25 | 1 | 0 | 0 | 1 | 0 | 0 | 0 | 0 | 0 | 2 | 1 |
| SEVERE-907 | 68 | 0 | 0 | 0 | 1 | 0 | 0 | 0 | 1 | 0 | 2 | 1 |
| SEVERE-908 | 36 | 0 | 0 | 1 | 0 | 0 | 0 | 0 | 1 | 0 | 3 | 1 |
| SEVERE-910 | 92 | 1 | 1 | 0 | 0 | 1 | 0 | 1 | 0 | 0 | 2 | 1 |
| SEVERE-911 | 70 | 1 | 1 | 1 | 0 | 1 | 0 | 1 | 0 | 0 | 3 | 2 |
| SEVERE-912 | 61 | 1 | 0 | 0 | 0 | 0 | 0 | 0 | 0 | 0 | 1 | 1 |
| SEVERE-913 | 60 | 1 | 0 | 1 | 0 | 0 | 1 | 0 | 0 | 0 | 2 | 1 |
| SEVERE-915 | 62 | 1 | 0 | 0 | 0 | 0 | 0 | 1 | 0 | 0 | 2 | 2 |
| SEVERE-916 | 79 | 1 | 1 | 0 | 1 | 1 | 0 | 0 | 0 | 1 | 2 | 2 |
| SEVERE-919 | 73 | 1 | 1 | 1 | 0 | 0 | 0 | 1 | 0 | 0 | 2 | 2 |
| SEVERE-922 | 75 | 1 | 1 | 0 | 0 | 0 | 1 | 0 | 0 | 0 | 1 | 2 |
| SEVERE-923 | 49 | 0 | 1 | 1 | 0 | 0 | 0 | 0 | 0 | 0 | 3 | 1 |
| SEVERE-925 | 71 | 1 | 0 | 0 | 0 | 0 | 0 | 0 | 0 | 0 | 1 | 1 |
| SEVERE-930 | 67 | 1 | 0 | 0 | 0 | 0 | 1 | 0 | 0 | 0 | 1 | 1 |
| SEVERE-933 | 70 | 1 | 1 | 1 | 0 | 1 | 0 | 0 | 0 | 0 | 3 | 2 |
| SEVERE-936 | 80 | 0 | 0 | 0 | 1 | 1 | 0 | 0 | 0 | 1 | 2 | 1 |
| SEVERE-937 | 59 | 0 | 1 | 1 | 0 | 0 | 1 | 0 | 0 | 0 | 3 | 1 |
| SEVERE-938 | 77 | 0 | 1 | 0 | 1 | 0 | 0 | 0 | 0 | 1 | 2 | 1 |
| SEVERE-941 | 33 | 1 | 0 | 1 | 0 | 0 | 1 | 0 | 0 | 0 | 2 | 2 |
| SEVERE-943 | 67 | 0 | 0 | 0 | 0 | 0 | 0 | 0 | 0 | 0 | 2 | 1 |
| SEVERE-945 | 55 | 0 | 1 | 1 | 1 | 0 | 0 | 0 | 0 | 1 | 3 | 1 |
| SEVERE-946 | 76 | 1 | 1 | 0 | 0 | 0 | 0 | 0 | 0 | 0 | 3 | 1 |
| SEVERE-947 | 81 | 1 | 1 | 1 | 0 | 1 | 0 | 0 | 0 | 0 | 2 | 2 |
| SEVERE-949 | 49 | 1 | 0 | 0 | 0 | 0 | 0 | 0 | 0 | 0 | 2 | 2 |
| SEVERE-950 | 53 | 0 | 0 | 0 | 0 | 1 | 0 | 0 | 0 | 0 | 1 | 1 |
| SEVERE-951 | 61 | 0 | 1 | 1 | 0 | 0 | 0 | 0 | 1 | 0 | 3 | 1 |
| SEVERE-952 | 64 | 1 | 1 | 0 | 0 | 0 | 0 | 0 | 0 | 0 | 2 | 1 |
| SEVERE-953 | 35 | 0 | 1 | 1 | 1 | 0 | 1 | 0 | 0 | 0 | 1 | 1 |
| SEVERE-955 | 53 | 0 | 1 | 1 | 0 | 0 | 0 | 0 | 0 | 0 | 2 | 3 |
| SEVERE-956 | 53 | 1 | 1 | 1 | 1 | 0 | 0 | 0 | 1 | 1 | 2 | 2 |
| SEVERE-959 | 75 | 0 | 1 | 0 | 0 | 1 | 0 | 0 | 0 | 0 | 2 | 1 |
| SEVERE-960 | 69 | 0 | 1 | 0 | 1 | 0 | 0 | 0 | 0 | 0 | 1 | 1 |
| SEVERE-961 | 75 | 1 | 0 | 0 | 0 | 1 | 1 | 1 | 0 | 0 | 3 | 1 |
| SEVERE-963 | 33 | 0 | 0 | 0 | 0 | 0 | 1 | 1 | 0 | 0 | 2 | 1 |
| SEVERE-964 | 59 | 1 | 1 | 1 | 1 | 0 | 1 | 0 | 0 | 0 | 2 | 2 |
| SEVERE-965 | 49 | 0 | 0 | 1 | 0 | 0 | 0 | 0 | 0 | 0 | 3 | 1 |
| SEVERE-966 | 62 | 0 | 1 | 0 | 1 | 0 | 0 | 0 | 0 | 0 | 2 | 1 |
| SEVERE-967 | 53 | 1 | 0 | 1 | 0 | 1 | 0 | 1 | 0 | 0 | 1 | 1 |
| SEVERE-968 | 78 | 1 | 1 | 0 | 0 | 0 | 0 | 0 | 0 | 0 | 1 | 2 |
| SEVERE-969 | 74 | 0 | 1 | 1 | 0 | 0 | 0 | 0 | 0 | 0 | 1 | 3 |
| SEVERE-970 | 61 | 1 | 0 | 0 | 1 | 0 | 0 | 0 | 0 | 1 | 1 | 1 |
| SEVERE-971 | 73 | 0 | 0 | 0 | 1 | 1 | 0 | 0 | 0 | 0 | 2 | 3 |
| SEVERE-972 | 55 | 1 | 1 | 1 | 1 | 1 | 0 | 0 | 0 | 1 | 3 | 1 |
| SEVERE-986 | 55 | 0 | 1 | 0 | 0 | 0 | 0 | 0 | 0 | 0 | 2 | 1 |
| SEVERE-992 | 89 | 0 | 1 | 0 | 0 | 0 | 0 | 0 | 0 | 0 | 1 | 1 |

RSDs – Respiratory System Diseases. CVDs – Cardiovascular Diseases. NDDs – Neuropsychiatric Diseases or Disorders. ACKDs – Acute or Chronic Kidney Diseases. 1=No or Ausence; 0=Yes or Presence. ACE ID genotypes (II=1; ID=2; DD=3); ACE2 G8790A genotypes (GG=1, GA=2, AA=3 for female; G=1 and A=3 for male).
